# Extracting Value from Marine and Microbial Natural Product Artifacts and Chemical Reactivity

**DOI:** 10.3390/md24010005

**Published:** 2025-12-20

**Authors:** Mark S. Butler, Robert J. Capon

**Affiliations:** Centre for Chemistry and Drug Discovery, Institute for Molecular Bioscience, The University of Queensland, Brisbane 4072, Australia

**Keywords:** marine natural products, marine bioproducts, artifacts, chemical reactivity, chemical stability, drug discovery

## Abstract

Natural products are and continue to be a remarkable resource, rich in structural diversity, and endowed with valuable chemical and biological properties that have advanced both science and society. Some natural products, especially those from marine organisms, are chemically reactive, and during extraction and handling can partially or totally transform into artifacts. All too often overlooked or mischaracterised as natural products, artifacts can be invaluable indicators of a uniquely evolved and primed chemical space, with enhanced chemical and biological properties highly prized for drug discovery. To demonstrate this potential, we review a wide selection of marine and microbial case studies, revealing the factors that initiate artifact formation (e.g., solvents, heat, pH, light and air oxidation) and commenting on the mechanisms behind artifact formation. We conclude with reflections on how to recognise and control artifact formation, and how to exploit knowledge of artifacts as a window into unique regions of natural product chemical space—to better inform the development of future marine bioproducts.

## 1. Introduction

Since the emergence of the first single-cell organisms, natural products have co-evolved with all kingdoms of life to deliver a multitude of survival advantages—defending against predators/competitors, immobilising/killing prey, facilitating reproduction, and articulating intra/interspecies communication and ecological behaviour. With the advent of modern science, knowledge of the molecular structures and properties of natural products has provided valuable insights into what is often synthetically challenging/inaccessible regions of chemical space, populated by diverse structures featuring complex carbo/heterocyclic scaffolds, functionality and chirality. Many natural products possess potent and selective ecological, chemical and biological properties, knowledge of which has informed our understanding of living systems, inspiring many of the world’s most successful drugs, agrochemicals and biomaterials, and fuelling a revolution in industry, commerce, healthcare and agriculture. Notwithstanding the extraordinary impact that natural products have had on science and society, the innate chemical reactivity of some natural products both defines their uniqueness and potential, while simultaneously presenting a technical challenge. For example, some natural products partially or completely transform into artifacts during extraction, isolation, handling and/or storage. When these transformations go unnoticed, it represents a lost opportunity, as we forgo insights into molecular structures and chemical reactivity that might otherwise inform biosynthetic investigations, biomimetic syntheses, structure–activity relationships (SARs) and mechanisms of action, and ultimately advance drug discovery and development.

For the most part, recent reviews in the area of natural product artifacts present artifacts more as a problem than an opportunity, restricting discussion to analytical approaches for detection, and even on occasion confusing the concept of artifacts with that of contaminants (e.g., plasticizers, antioxidants, etc.) [1,2,3,4,5,6]. In an alternate view presented in a 2020 review [7], Capon (the corresponding author) used a selection of case studies in marine natural product chemistry to demonstrate how a willingness to detect and understand natural product artifacts could return significant knowledge dividends.

This current review builds on and extends that value-added proposition, addressing the inter-connected concepts of natural product artifacts and innate chemical reactivity, as illustrated via a new set of marine and microbial natural product case studies (Table 1). The inclusion of examples from non-marine microbes serves to widen the natural product/artifact knowledge base to include more diverse and mechanistically important case studies, while also recognising the significant overlap between non-marine and marine microbial natural products science. The current review also pays particular attention to the factors that initiate, and the underlying mechanisms behind, artifact formation (Section 2, Section 3, Section 4, Section 5, Section 6, Section 7, Section 8 and Section 9). For example, given the critical and nearly ubiquitous role of solvents in natural products science, we survey the risks posed by different solvents in artifact formation. We also explore the influence of heat, pH, light and air, and the propensity of certain classes of natural products to undergo structural diversification through the likes of acetal/ketal equilibration, *trans*-esterification and epimerisation, and introduce two new concepts to the natural product/artifact discourse.

*Cryptic natural products* (see Section 10)—Defined as those natural products endowed with such high levels of chemical reactivity as to preclude detection and isolation. The “unknown unknowns” of the natural product world, cryptic natural products are often only hinted at by the artifacts they leave behind. For the observant researcher, however, perseverance in pursuing the unknown can be rewarded by a hidden cache of knowledge.

*Bioassay biotransformation* (see Section 11)—We draw attention to a largely overlooked phenomenon, where some chemically reactive natural products applied to enzyme/cell-based bioassays undergo in situ biotransformation, compromising bioassay data analysis and undermining structure–activity relationship analyses, and knowledge relating to pharmacophores and mechanisms-of-action.

In the Conclusion (Section 12), we reflect on the case studies outlined in the review, and our own experiences, to propose best-practice workflows for detecting and mitigating artifact formation, and for recognising and leveraging knowledge of artifacts.

Throughout the review, we make extensive use of figures to illustrate molecular diversity and relationships between natural products and artifacts, and the mechanisms that underpin these relationships. Where figures feature arrows of different colours, these denote alternate mechanistic pathways, and where atoms, bonds or ring systems are shaded with varying colours, these are designed to help focus attention on chemically reactive key functionality, and/or structural features that differentiate natural products from artifacts.

Prior to exploring case studies, it is worthwhile taking a moment to first agree on a workable definition that answers the question of *“What is a natural product?”*, in order to arrive at an acceptable definition of *“What is an artifact?”* Curiously, this is not as straightforward as one might imagine. Although the term *“natural product”* defines an entire field of science and is in common use across multiple disciplines, it is difficult to find a clear and unambiguous definition of “*What is a natural product?*”. For the majority of the previous century, the chemistry of life was largely viewed as a binary divide between primary metabolites (essential for life), and secondary metabolites (important, but not essential for life). The term secondary metabolite became synonymous with natural product, so much so that for some it morphed into a circular definition, where a natural product was defined as a secondary metabolite, and a secondary metabolite was defined as a natural product. Over time, as more knowledge became available, this binary divide did not fare well. For example, when a prospective new natural product is first discovered (i.e., isolated and identified), it is typically the case that we do not know its ecology/biology, let alone whether it is important or essential to life. Even these latter two criteria are themselves vague—at what point does *important for life* transition to *essential to life*? Think of hormones, pheromones, venoms, or pigments. Nevertheless, even in the absence of a clear definition, legions of new natural products are published in the scientific literature each year, presumably on the basis that researchers instinctively know a natural product when they see one. Putting aside the limitations of the primary versus secondary metabolism duopoly, which has arguably outlived its usefulness, for the purpose of this review we propose the following working definitions.

*A natural product*—a chemical that originates from and can be detected in a fresh unfractionated extract of a source organism, provided the process of extraction and/or detection does not initiate a chemical transformation that is solely responsible for producing the chemical.

The latter proviso seeks to account for those highly reactive (cryptic) natural products that rapidly transform into artifacts during extraction (see Section 10), such that the natural product remains invisible and only the artifact is detected in the extract and erroneously attributed natural product status.

*An artifact*—a natural product that has undergone a chemical transformation during extraction, handling, storage and/or analysis.

As a codicil, natural product and artifact status are not necessarily mutually exclusive. This may seem counter-intuitive, but consider, for example, a compound that meets the definition of a natural product but that can *also* be obtained by the transformation of a co-metabolite during extraction and handling. This duality reflects the situation where chemical reactivity provides both a path for (i) non-enzyme-mediated natural product formation and diversification in situ within the source organism and (ii) artifact formation during or post extraction.

## 2. Solvents

To isolate, identify and study natural products first requires extraction from the producing organism (i.e., a microbial fermentation, or a macro marine organism such as a sponge, alga, tunicate, etc.), followed by fractionation, spectroscopic, chemical and biological data acquisition and analysis, as well as handling and storage. All these processes require exposure to solvents, leaving open the possibility of forming artifacts. As is evident from the case studies outlined below, while all solvents bring with them some risk of forming artifacts, some solvents clearly present higher risk than others.

### 2.1. DMSO

#### Migrastatins/Dorrigocins (Figure 1)

The glutarimide polyketides, *iso*-migrastatin (**2.1**), migrastatin (**2.2**) and dorrigocins A (**2.3**) and B (**2.4**), were initially isolated as co-metabolites of *Streptomyces platensis* (NRRL 18993) [8,9]. Subsequent studies identified an additional analogue, 13-*epi*-dorrigocin A (**2.5**), and revealed that **2.1** alone was a natural product, with **2.2** to **2.5** produced when **2.1** was stored in DMSO-H_2_O [10]. These latter transformations can be rationalised as H_2_O addition to C-13 with concomitant double bond migration and opening of the macrolactone to generate *E* and *Z* Δ^11^ isomers: the *E* isomer leads to dorrigocin A (**2.3**), 13-*epi*-dorrigocin A (**2.5**) and dorrigocin B (**2.4**) and the *Z* isomer undergoes relactonisation to yield migrastatin (**2.2**). Knowledge of this chemical reactivity was later exploited to produce a library of *iso*-migrastatin congeners [11], while further studies demonstrated a direct thermally induced [3]-sigmatropic rearrangement of **2.1** to **2.2**, paving the way for new analogues [12].

**Figure 1:**
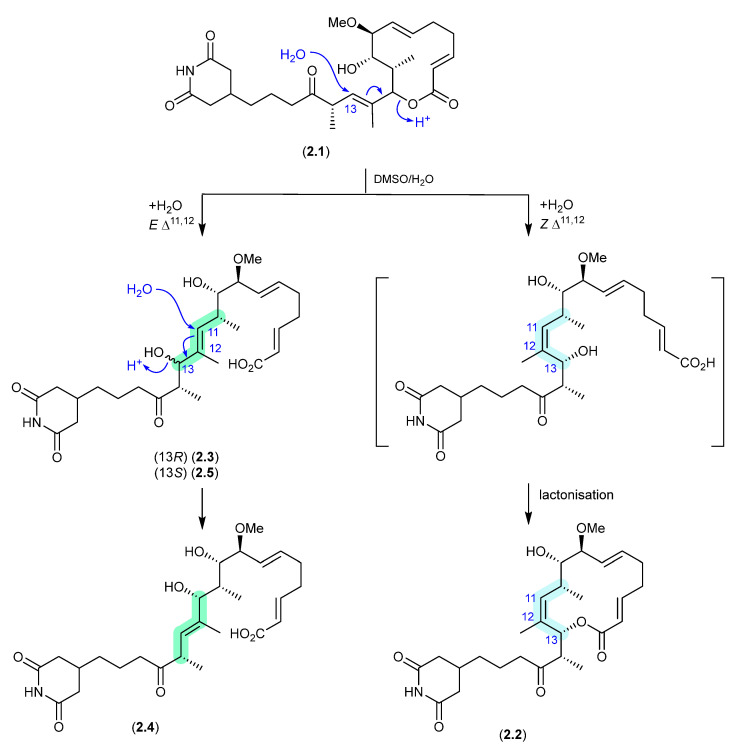
Migrastatins/Dorrigocins.

#### Discorhabdins (Figure 2)

On handling in DMSO-*d*_6_, three pyrroloiminoquinone discorhabdins, H (**2.6**), L (**2.7**) and B (**2.8**), isolated from the New Zealand marine sponge *Latrunculia kaakaariki*, transformed into the trideuteromethyl artifacts **2.9**–**2.11** [13]. Investigations into the mechanism behind this transformation suggested it occurred during recovery of NMR (DMSO*-d*_6_) samples in the presence of MeOH, H_2_O and trifluoroacetic acid (TFA) (with all three of these solvents being essential for efficient incorporation of the CD_3_ moiety). ICP-MS revealed trace levels of iron present in both the DMSO-*d*_6_ and TFA, presumably initiating OH radicals via a Fenton reaction.

**Figure 2:**
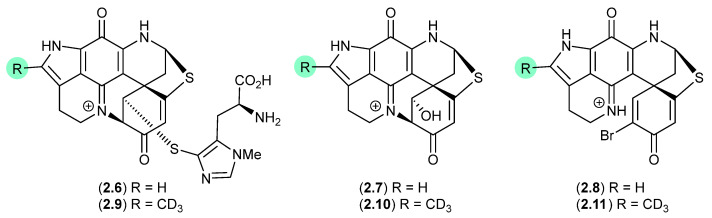
Discorhabdins.

#### Dendrillic Acids (Figure 3)

On handling in DMSO-*d*_6_, the spongian norditerpenoid dendrillic acids A (**2.12**) and B (**2.13**), isolated from the Western Australian marine sponge *Dendrilla* sp., readily equilibrated at room temperature (r.t.) to an epimeric mixture (**2.12**/**2.13**) [14]. This epimerisation likely proceeds via enolization of the lactam to an achiral pyrrolo intermediate, as supported by partial incorporation of deuterium into the γ-lactam methine when 5% D_2_O was added to the DMSO-*d*_6_ solution.

**Figure 3:**
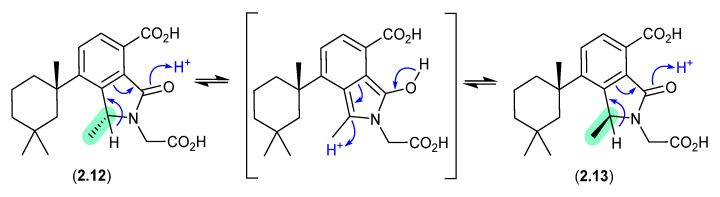
Dendrillic Acids.

#### Methylsulfonated Polyketides (Figure 4)

Supplementation of a fermentation of a mangrove-derived fungus *Neosartorya udagawae* HDN13-313 with a DMSO solution of the DNA methyltransferase inhibitor 5-azacytidine yielded the methylsulfonylated artifacts **2.14** and neosartoryone A (**2.15**) [15]. Investigating this transformation revealed that a fermentation supplemented with DMSO alone also produced **2.14**, which suggested that the methylsulfonyl moiety was derived from DMSO. In support of this hypothesis, fermentation in the presence of DMSO-*d*_6_ returned the corresponding deuterated analogues of both **2.14** and **2.15**.

**Figure 4:**
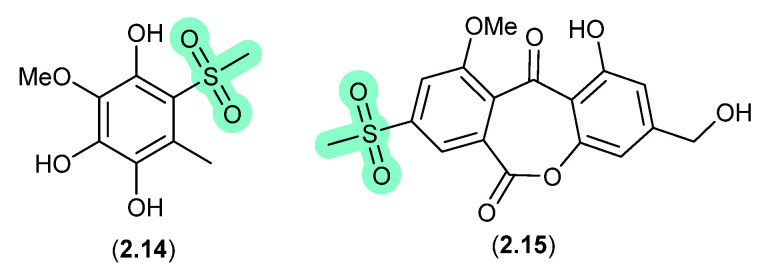
Methylsulfonated Polyketides.

#### Cerulenin (Figure 5)

Cerulenin (**2.16**), originally reported in the middle of the previous century from the fungus *Cephalosporium caerulens* KF-140, was the first reported natural fatty acid synthase inhibitor, with anticancer, antifungal and anti-obesity potential. In protic solvents, **2.16** undergoes intramolecular 5-*exo-trig* cyclization to generate cerulenin hydroxylactams **2.17** and **2.18**. For example, a recent report confirmed that an NMR (DMSO-*d*_6_) sample exists almost entirely in the acyclic form (**2.16**), whereas LC-MS in MeOH/H_2_O/HCO_2_H revealed a three-component mixture (**2.16**–**2.18**) [16].

**Figure 5:**
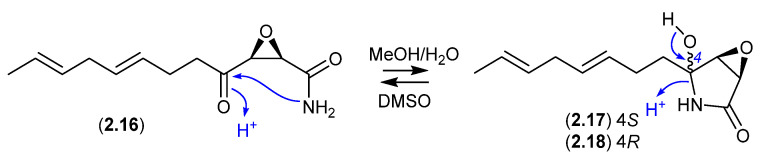
Cerulenin.

#### Bisanthraquinones (Figure 6)

An investigation into a *Streptomyces* sp. isolated from a cyanobacterium associated with the Puerto Rican tunicate *Ecteinascidia turbinata* yielded two antibacterial bisanthraquinones, **2.19** and **2.20** [17]. During long NMR (DMSO-*d*_6_) acquisitions, both these metabolites degraded to a single common dehydration artifact, **2.21**. Partial conversion of **2.19** to **2.21** was also achieved by allowing **2.19** to stand at r.t. in DMSO for several days. Of note, the artifact **2.21** was 220-fold more active against methicillin-resistant *Staphylococcus aureus* (MRSA) than the parent natural product **2.19**.

**Figure 6:**
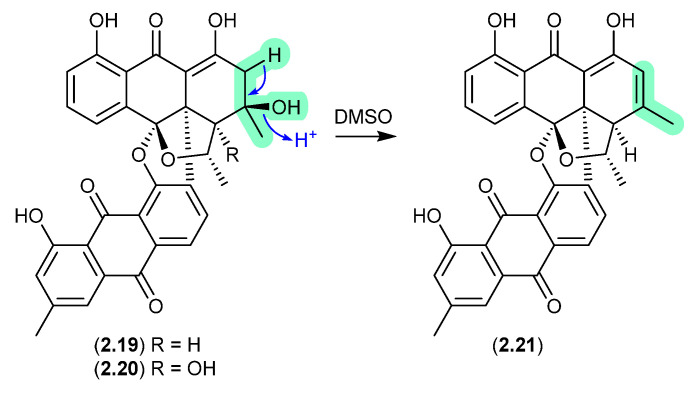
Bisanthraquinones.

#### Glyclauxins (Figure 7)

The Australian wasp nest-derived fungus *Talaromyces* sp. CMB-MW102 yielded the known duclauxin (**2.22**) (see Section 4.3) and a series of 1-deoxy-d-glucosamine adducts, glyclauxins A–E [18]. In an effort to understand the biosynthetic relationship between duclauxin and glyclauxins, and implement a biomimetic synthesis, an r.t. DMSO solution of **2.22** and synthetic 1-deoxy-d-glucosamine underwent quantitative conversion to glyclauxin B (**2.24**), while MeOH solutions of glyclauxins C (**2.25**) and D (**2.26**) underwent rapid and quantitative transformation to glyclauxins B (**2.24**) and A (**2.23**), respectively. Despite the ease of these latter transformations, both **2.23** and **2.24** were detected in fresh unfractionated culture extracts—attesting to their dual status as both natural products and artifacts (see Section 12).

**Figure 7:**
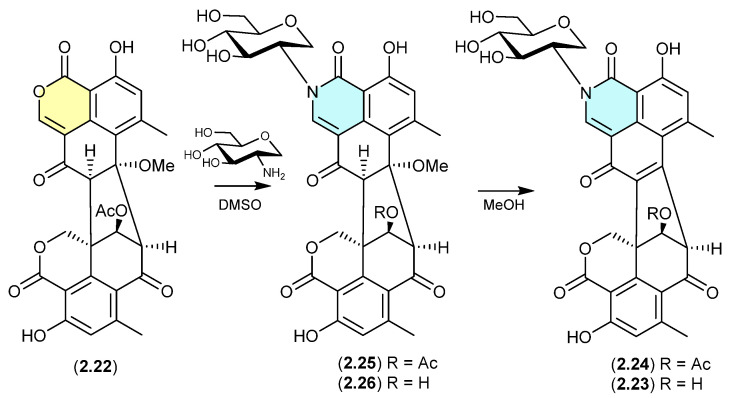
Glyclauxins.

#### Aculeaxanthones/Chrysoxanthones (Figure 8)

Aculeaxanthone B (**2.27**), from the marine-derived fungus *Aspergillus aculeatinus* WHUF0198, undergoes a retro-oxa-Michael equilibration in DMSO-*d*_6_ to the regioisomer, chrysoaxanthone B (**2.28**) [19]. As **2.28** was itself reported as a natural product from the sponge-derived *Penicillium chrysogenum* HLS111 [20], this raises an interesting question—“Is one regioisomer an artifact, and if so, which one?”

**Figure 8:**
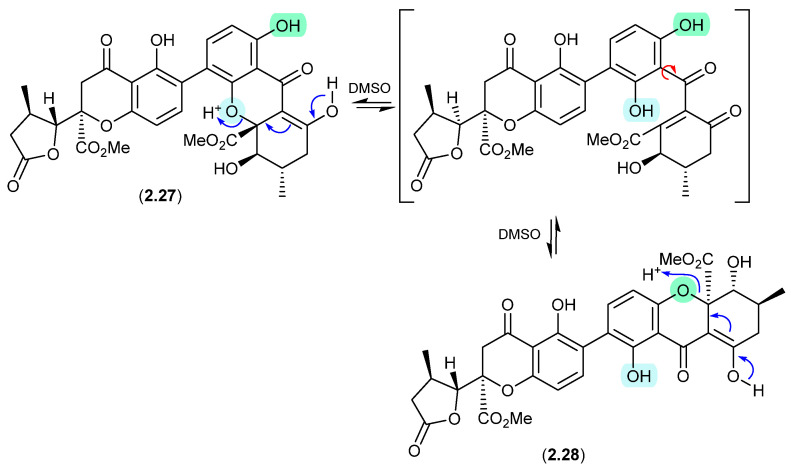
Aculeaxanthones/Chrysoxanthones.

#### Versixanthones (Figure 9)

Similar DMSO-mediated retro-oxa-Michael equilibrations were reported in 2015 between versixanthones A (**2.29**) and D (**2.30**), and versixanthones B (**2.31**) and C (**2.32**), which were isolated from the marine-derived fungus *Aspergillus versicolor* HDN100.9 [21].

**Figure 9:**
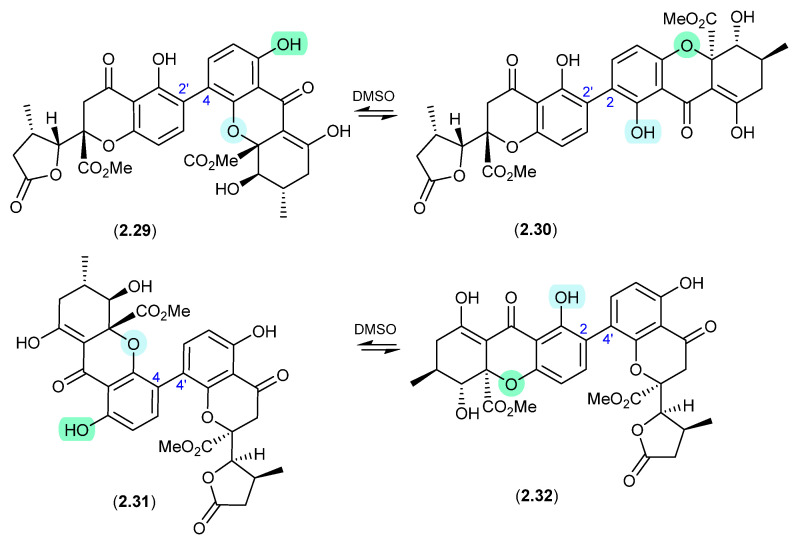
Versixanthones.

#### Secalonic Acid/Parnafungins (Figure 10 and Figure 11)

This solvent-mediated intramolecular retro-oxa-Michael equilibration was further demonstrated in studies of secalonic acid A (**2.33**) and its associated regioisomers [22,23], as well as in the relationship between parnafungin A, which is a mixture of the C-15a epimers parnafungins A1 (**2.34**) and A2 (**2.35**), and parnafungin B, a mixture of the C-15a epimers parnafungins B1 (**2.36**) and B2 (**2.37**) [24].

**Figure 10:**
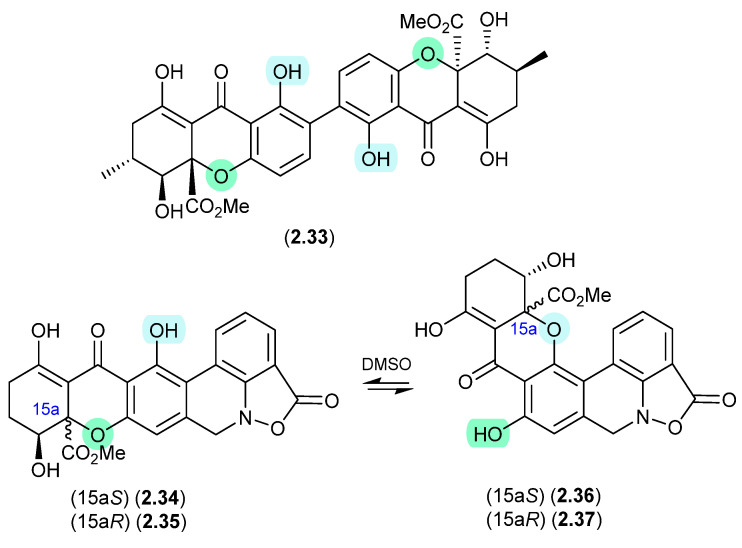
Secalonic Acid/Parnafungins.

Of note, the chemical reactivity of the tetrahydroxanthone–chromanone core common across **2.33**–**2.37** is not restricted to the intramolecular retro-oxa-Michael equilibrations outlined above. For example, while DMSO mediated an intra-molecular retro-oxa-Michael equilibration between chrysoxanthones E (**2.38**) and G (**2.39**), exposure of **2.38** to H_2_O facilitated hydrolysis of ring A to chrysoxanthone O (**2.40**), while the addition of MeOH extended this to include the methanolysis product chrysoxanthone P (**2.41**) [25]. Unsurprisingly, the opening of ring A removed the ability to undertake retro-oxa-Michael equilibration. With DMSO, MeOH and H_2_O being common extraction, purification and handling solvents, the ease with which these r.t. transformations take place raises the likelihood that handling artifacts have been misidentified as natural products. It also challenges the veracity of bioactivity data and the ability to assign SAR, where analytes can transform during bioassays (see Section 11).

**Figure 11:**
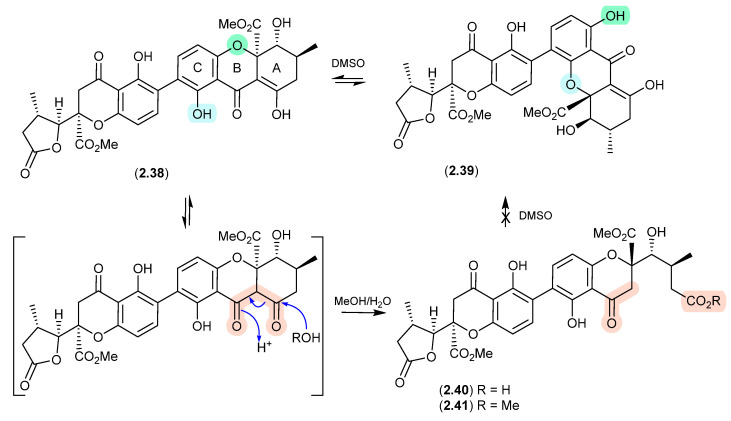
Chrysoxanthones.

### 2.2. Pyridine

#### Acremoxanthones/Acremonidins (Figure 12)

An unidentified fungus of the order Hypocreales (MSX 17022) yielded two new xanthone–anthraquinone heterodimers, acremoxanthones C (**2.42**) and D (**2.43**), and the closely related known metabolites acremonidins C (**2.44**) and A (**2.45**) [26]. Interestingly, attempts at preparing Mosher esters revealed that **2.43** was unstable to pyridine. Indeed, when exposed to pyridine alone at r.t. for 4.5 h, **2.43** underwent complete conversion to **2.45**. As noted above (Section 2.1), such a transformation would be expected to occur under neutral conditions in DMSO.

**Figure 12:**
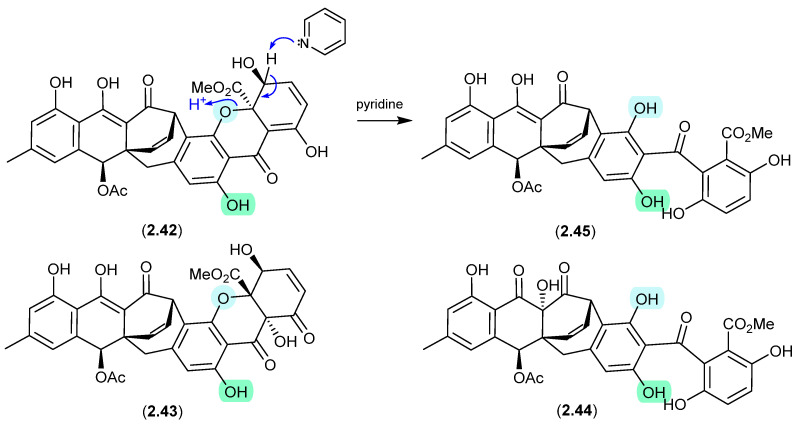
Acremoxanthones/Acremonidins.

### 2.3. Methanol

#### Brevianamides (Figure 13 and Figure 14)

The Lake Michigan deep-water sediment-derived fungus *Penicillium* sp. 5-PBA-2 yielded two diketopiperazines, brevianamides E1 (**2.46**) and E2 (**2.47**), along with known analogues, including brevianamide E (**2.48**) [27]. Of note, **2.46** and **2.47** rapidly equilibrated (1:9 ratio) in acidic HPLC solvents (MeCN/H_2_O with either 0.1% formic acid or 0.1% TFA), while prolonged exposure of the equilibrating mixture to acidic MeOH (0.1% TFA) yielded the methyl ether **2.49**, as well as the two rearranged products **2.50** and **2.51**. It is proposed that the latter are formed by a reaction with formaldehyde present as an impurity in commercial MeOH—confirmed by addition of 2,4-dinitrophenylhydrazine to commercial MeOH and the detection of formaldehyde 2,4-dinitrophenylhydrazone.

A comparison of experimental and calculated ECD spectra (with **2.47**) led to structure revisions for known diketopiperazines featuring a rare 11-oxy moiety, including notoamides K (**2.52**) and P (**2.53**), and asperversiamide L (**2.54**) [27].

**Figure 13:**
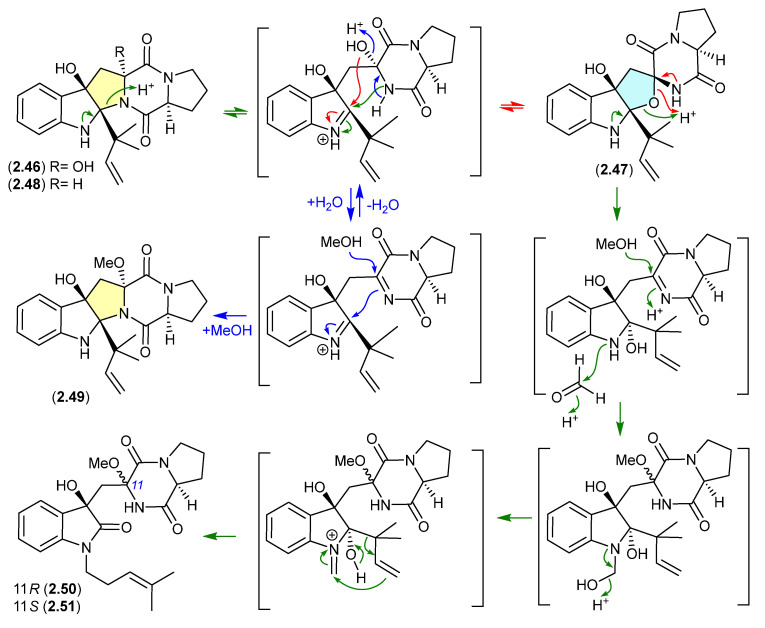
Brevianamides.

**Figure 14:**
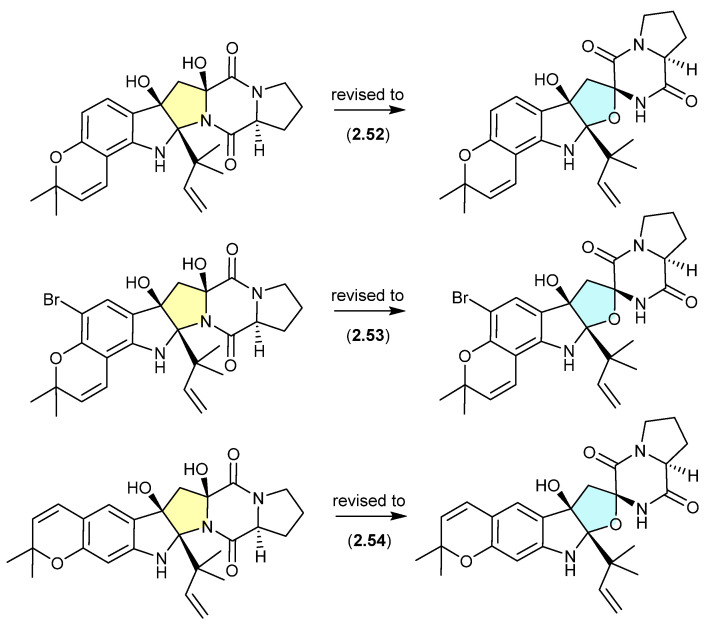
Notoamides/asperversiamides.

#### Talaronins (Figure 15)

The Chinese mangrove-derived fungus *Talaromyces* sp. WHUF0362 yielded a range of polyketides, including the new depsidone dimethylacetals, talaronins A (**2.55**) and B (**2.56**), the new depsidone benzaldehyde talaronin D (**2.57**) and the known purpactin C′ (**2.58**) [28]. Although the dimethylacetals **2.55** and **2.56** are designated as natural products, the extensive use of MeOH eluant across Sephadex LH-20 and HPLC makes it far more probable that the dimethylacetals are methanolysis artifacts of **2.57** and **2.58**, respectively. Other examples of natural product aldehydes transforming in MeOH to dimethyl acetals include the colletotrichalactones from the Korean leaf-derived endophytic fungus *Colletotrichum* sp. JS-0361 [29], cladosporisteroids from the Chinese marine sponge-derived fungus *Cladosporium* sp. SCSIO41007 [30] and the talaromycins from the South China Sea gorgonian-derived fungus *Talaromyces* sp. (see Section 5.3) [31].

**Figure 15:**
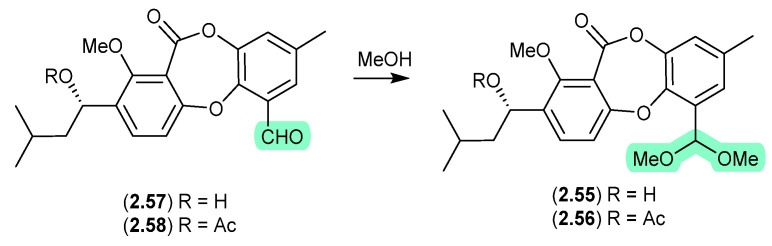
Talaronins.

#### Pyrasplorins (Figure 16)

The mangrove-derived fungus *Aspergillus versicolor* yielded an array of pyrazinopyrimidine alkaloids, pyrasplorins A–C (**2.59**–**2.61**) [32]. On handling in MeOH, **2.60** underwent ring opening to the artifact **2.62**; however, it is likely that the ring opening was mediated by H_2_O present in the MeOH). Although the authors make no mention of a plausible dehydration pathway from the artifact **2.62** to **2.61**, it raises the prospect that the latter is also an artifact.

**Figure 16:**
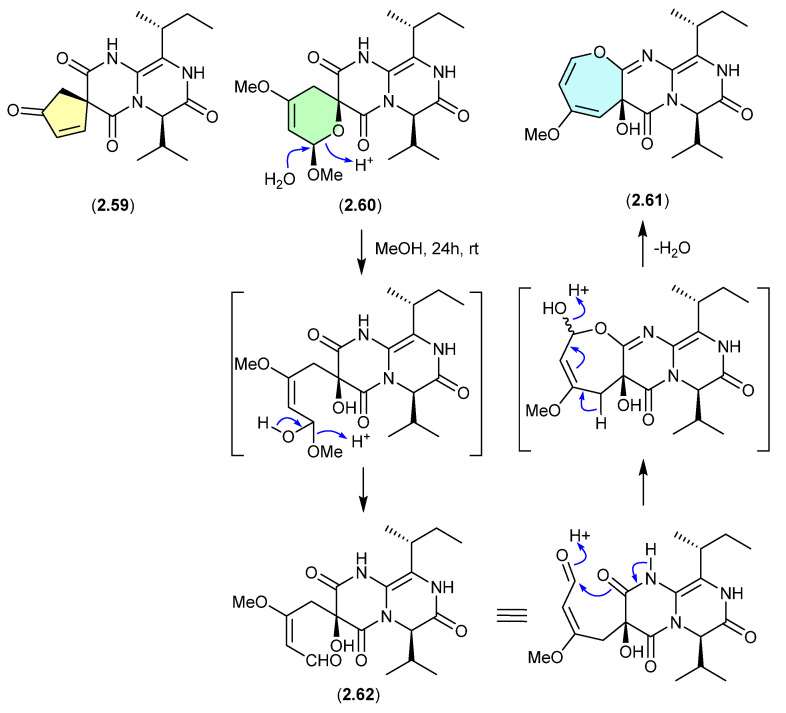
Pyrasplorins.

#### Penicipyridones (Figure 17)

The marine mangrove plant-derived fungus *Penicillium oxalicum* QDU1 yielded 11 new pyridine alkaloids, exemplified by penicipyridones A–C (**2.63**–**2.65**) [33]. Indicative of their chemical reactivity, an acidic solution of **2.63** (MeCN/H_2_O or MeOH/H_2_O, with 0.1% formic acid) transformed into **2.64** over 3 days, with trace amounts of **2.65**. After 14 d, acidic MeOH solutions of any one of **2.63**–**2.65** equilibrated to an 8:7:1 mixture. This reactivity reveals a biomimetic path to adding unnatural nucleophiles at C-4.

**Figure 17:**
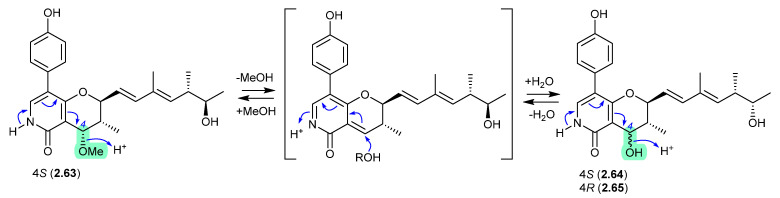
Penicipyridones.

#### Variacins (Figure 18)

The benzopentathiepin varacin (**2.66**), isolated from a Fijian sample of the marine ascidian *Lissoclinum vareau*, exhibited potent cytotoxic properties against human colon carcinoma cells (HCT 116) [34]. Subsequently, **2.66** was reported along with three analogues, varacins A–C (**2.67**–**2.69**), from a Sea of Japan ascidian *Polycitor* sp. [35]. Interestingly, solutions of **2.66** or **2.67** (in MeOH, CH_2_Cl_2_ or pyridine) equilibrate to mixtures of **2.66**, **2.67** and S_8_ (**2.70**).

**Figure 18:**
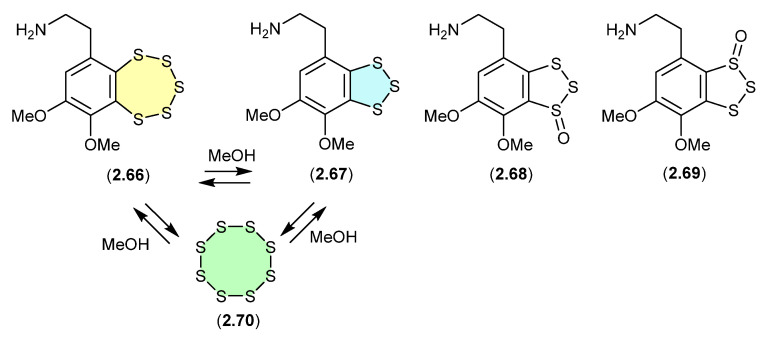
Variacins.

#### Epithiodiketopiperazines (Figure 19 and Figure 20)

The study of the fungal strain *Penicillium* sp. YE isolated from a Florida collection of a coral *Pseudodiploria strigosa* infected with coral black band disease yielded four new epithiodiketopiperazines, penigainamides A (**2.71**), B (**2.72**), C (**2.73**) and D (**2.74**), and five known analogues, adametizine A (**2.75**), FA2097 (**2.76**), outovirins A (**2.77**) and C (**2.78**) and pretrichodermamide C (**2.79**) [36]. Some of these proved unstable to handling in a range of solvents, leading to either contraction/expansion of the di, tri and tetra thioether ring and/or transformation between chlorohydrin and epoxide moieties. For example, exposure of (i) **2.75** to MeOH (1 wk) lead to partial conversion to **2.71**–**2.73** and **2.76**, and H_2_O (1 wk) to **2.72**, **2.76**, **2.78** and **2.79**; (ii) **2.71** to CDCl_3_ (1 h) lead to partial conversion to **2.74**; (iii) **2.72** to methanol-*d*_4_ (30 min) to **2.73**; and (iv) **2.78** to H_2_O (1 wk) to **2.77**, to acetone-*d*_6_ (30 min) to **2.74**, and to artificial sea water culture media (PDB) to **2.75**. It is proposed that the S ring expansions and contraction are caused by light-induced sulfur radical formation and subsequent desulfurisation and intermolecular disproportionation (see chetomins (Figure 68) [37]).

**Figure 19:**
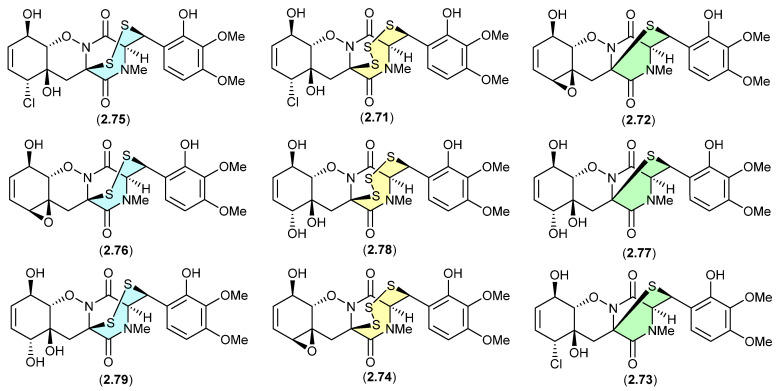
Epithiodiketopiperazines.

**Figure 20:**
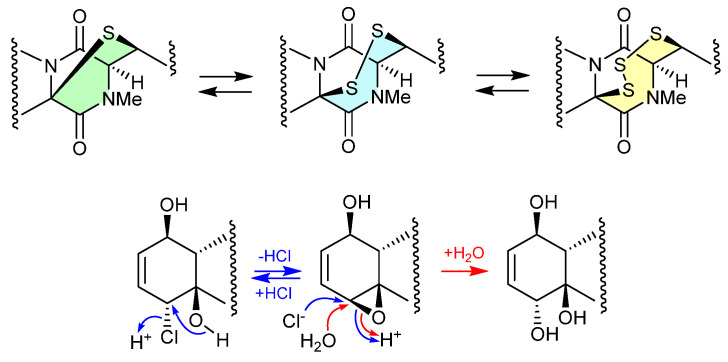
Epithiodiketopiperazines.

#### Eleutherobins/Caribaeoranes (Figure 21)

Eleutherobin (**2.80**), first reported in 1997 by Fenical et al. from a Western Australian sample of soft coral *Elutherobia* sp., exhibited exceptional activity as a microtubule-stabilising antimitotic agent that was comparable to paclitaxel [38]. Subsequently, Andersen et al. reported the re-isolation of **2.80** in 2000, along with the six new analogues, desmethyleleutherobin (**2.81**), desacetyleleutherobin (**2.82**), isoeleutherobin A (**2.83**), *Z*-eleutherobin (**2.84**), caribaeoside (**2.85**) and caribaeolin (**2.86**), from southern Caribbean samples of the octocoral *Erythropodium caribaeorum* [39]. In both studies, fractionation involved the use of silica gel (MeOH) chromatography, raising the possibility that the 4-methylacetals were handling artifacts. In a follow-up 2001 report, Andersen et al. described alternate solvent extractions of fresh collections of *E. caribaeorum*, with MeOH extraction returning the previously reported 4-methylacetals, and EtOH extraction returning the corresponding 4-ethylketals—revealing the true natural products in this class to be 4-hemiketals [40].

**Figure 21:**
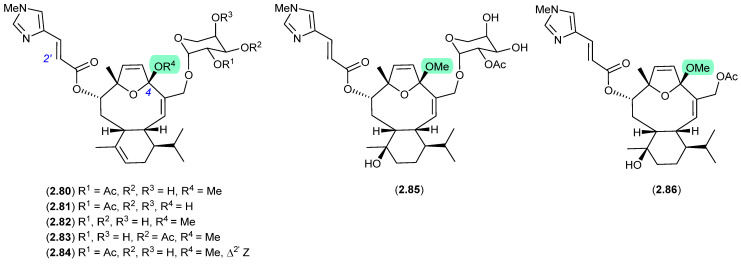
Eleutherobins/Caribaeoranes.

### 2.4. Acetone

#### Kutzneridines (Figure 22)

Genomic analysis of the Panama soil-derived actinobacterium *Kutzneria* sp. CA-103260 revealed a biosynthetic gene cluster (BGC) encoding for a putative lipopeptide—with heterologous expression in *Streptomyces coelicolor* M1152 yielding kutzneridine A (**2.87**), bearing an exotic *N*,*N*-acetonide moiety [41]. However, as acetone was used in the extraction process, it was determined that **2.87** was an artifact of a cryptic natural product **2.88** (detected but not isolated/characterised—see Section 10).

**Figure 22:**
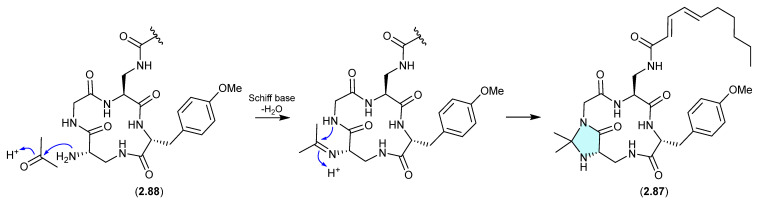
Kutzneridines.

#### Enamidonins/K97-0239A and B (Figure 23)

The *N*,*N*-acetonide-containing lipopeptide enamidonin (**2.89**) was first reported in 1995 from the soil-derived *Streptomyces* sp. 91-75 [42], and later in 2018 along with two new analogues, enamidonins B (**2.90**) and C (**2.91**) from a Korean soil-derived *Streptomyces* sp. KCB14A132 [43]. Likewise, the Japanese soil-derived *Streptomyces* sp. K97-0239 yielded the *N*,*N*-acetonide lipopeptides K97-0239A (**2.92**) and K97-0239AB (**2.93**) [44]. As all of the *N*,*N*-acetonides **2.89**–**2.93** were recovered by acetone extraction from their respective fermentations, it is likely all are handling artifacts.

**Figure 23:**
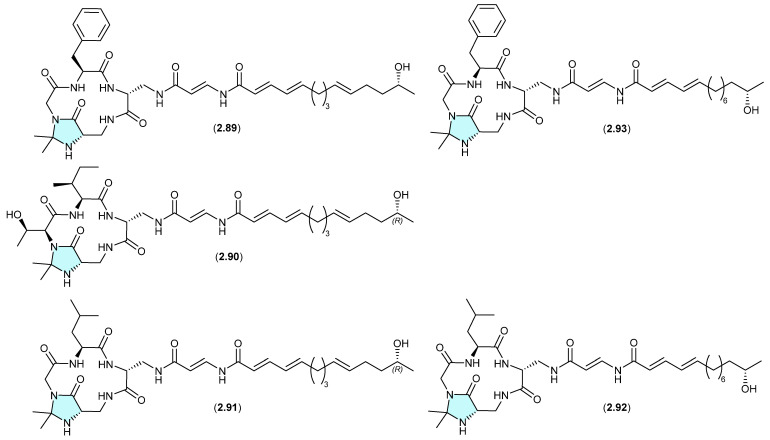
Enamidonins/K97-0239A and B.

#### Autucedines (Figure 24)

Genome mining of the deep-sea-derived *Streptomyces olivaceus* SCSIO T05 led to the discovery of five new enamidonin (**2.89**)-related lipopeptides [45]. Fermentations extracted with acetone produced three *N*,*N*-acetonide adducts, autucedines A (**2.94**), D (**2.95**) and E (**2.96**), while extraction with 2-butanone produced the analogous *N*,*N*-ethyl,methyl adducts autucedines B (**2.97**) and C (**2.98**). This study has taken advantage of this handling artifact reaction to produce new analogues.

**Figure 24:**
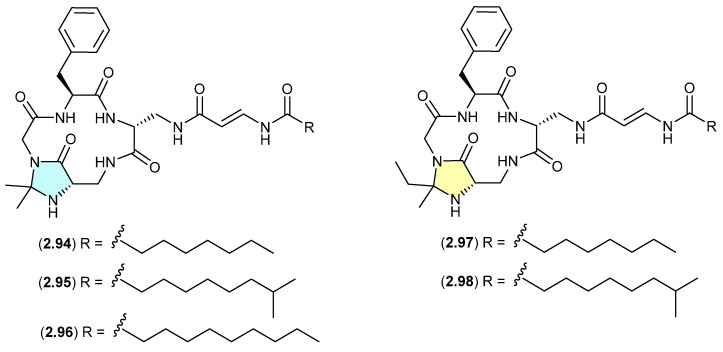
Autucedines.

#### Madurastatins (Figure 25)

The marine sponge-derived *Actinomadura* sp. WMMA-1423 yielded two new *N*,*N*-acetal siderophores, madurastatins D1 (**2.99**) and D2 (**2.100**), along with madurastatin C1 (**2.101**) [46]. As the fermentation was extracted with acetone, its likely **2.101** reacts with acetone to yield the *N*,*N*-acetonide artifact **2.100**, and with acetaldehyde—a common contaminant in commercial (especially recycled) acetone—to yield the artifact **2.99**. Presumably, *N*-methylation diminishes chemical reactivity, which is why on this occasion the precursor **2.101** survived extraction—unlike the case with **2.89**–**2.98**.

A 2024 study of the acetone extracts of a South African plant rhizosphere-derived *Actinomadura* sp., CA-135719, identified madurastatins H2 (**2.102**) and 33-*epi*-D (**2.103**) and reassigned absolute configurations across **2.99**–**2.103** [47]. Significantly, these authors concluded that the madurastatins **2.99**, **2.100**, **2.102** and **2.103** are in all probability acetone handling artifacts, although there could be some contribution from low levels of endogenous aldehydes. Of note, **2.102** is also likely an acid-mediated hydrolysis analogue (artifact) of **2.100** where the oxazoline ring is opened to a serine residue (see Section 4.2, serratiochelin).

**Figure 25:**
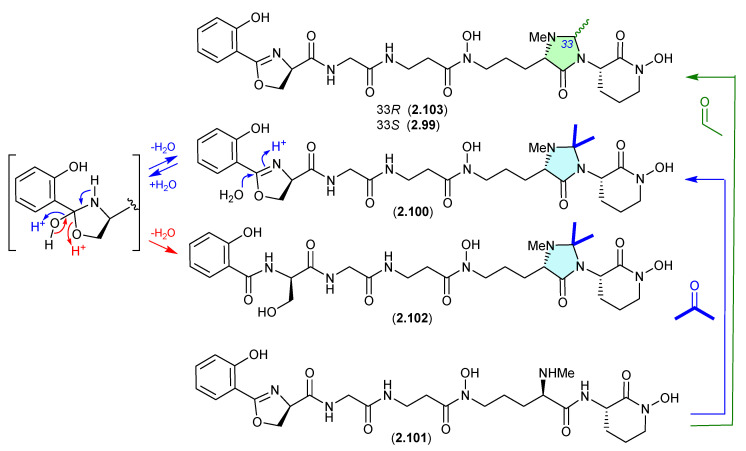
Madurastatins.

#### Drimanes (Figure 26)

The Okhotsk Sea sediment-derived fungus *Aspergillus ustus* KMM 4664 yielded a range of metabolites including the known drimane 12-hydroxyalbrassitriol (**2.104**) and the new acetonides **2.105** and **2.106** [48]. As the fungal fermentation was extracted with acetone (and chromatographed on silica gel), the acetonides are likely acetone artifacts.

**Figure 26:**
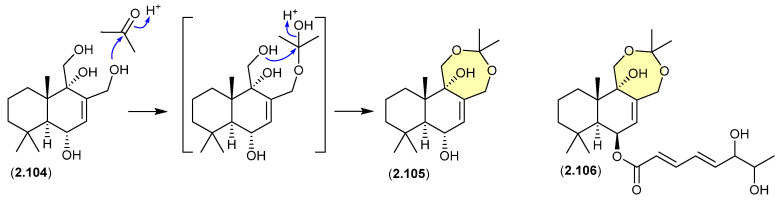
Drimanes.

#### Duclauxin/Verruculosins (Figure 27 and Figure 28)

A 2024 report by Rivera-Chávez et al. on the chemical reactivity of the fungal natural product duclauxin (**2.22**) (see Section 4.3) established that exposure to acetone led to the formation of verruculosin A (**2.107**) [49]. First reported from the acetone extract of the South China Sea soft coral-derived fungus *Talaromyces verruculosus* along with verruculosin B (**2.108**), **2.107** was originally designated as the first duclauxin*-like* natural product to possess an octacyclic skeleton [50]. Unlike the acetonide adducts described above (e.g., enamidonins, madurastatins), Rivera-Chávez et al. proposed that the transformation of **2.22** to **2.107** proceeds via an enamine-activated adduct of acetone (with a biogenetic amine present in the acetone extract). This hypothesis was confirmed by transformation of **2.22** to **2.107** in acetone supplemented with morpholine.

Building on this enamine-mediated mechanism, it is reasonable to propose a comparable transformation utilising acetaldehyde, a known contaminant in commercial acetone (see Section 2.4, madurastatins), followed by oxidation and methylation during silica gel (CH_2_Cl_2_/MeOH) chromatography (see Section 4.3), would see verruculosin B (**2.108**) also designated as an artifact of duclauxin (**2.22**).

**Figure 27:**
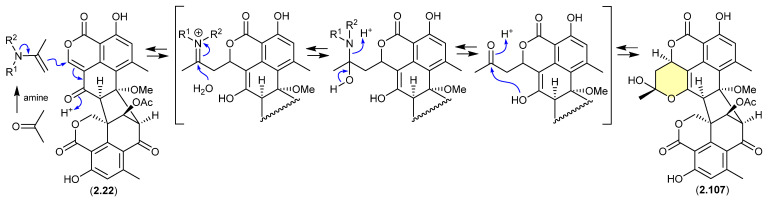
Duclauxin/Verruculosin A.

**Figure 28:**
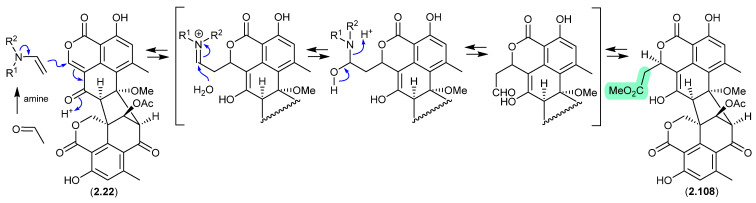
Duclauxin/Verruculosin B.

### 2.5. Acetonitrile

#### Talcarpones (Figure 29)

The Australian soil-derived fungus *Talaromyces johnpittii* MST-FP2594 yielded the new binaphthazarin talcarpones A (**2.109**) and B (**2.110**), along with the known monomeric naphthoquinone aureoquinone (**2.111**) [51]. When stored in aqueous MeCN at r.t. **2.110** transformed into **2.109** (presumably via H_2_O addition to an oxonium intermediate), and on heating (50 °C) further transformed into **2.111**. Conversely, at r.t. an acidic MeOH (5% TFA) solution of **2.109** transformed into **2.110** (presumably via MeOH addition to an oxonium intermediate). As the acetone extract of *T. johnpittii* was subjected to silica gel (CH_2_Cl_2_/MeOH) chromatography (see Section 4.3), it seems likely **2.110** is a methanolysis artifact of **2.109**.

**Figure 29:**
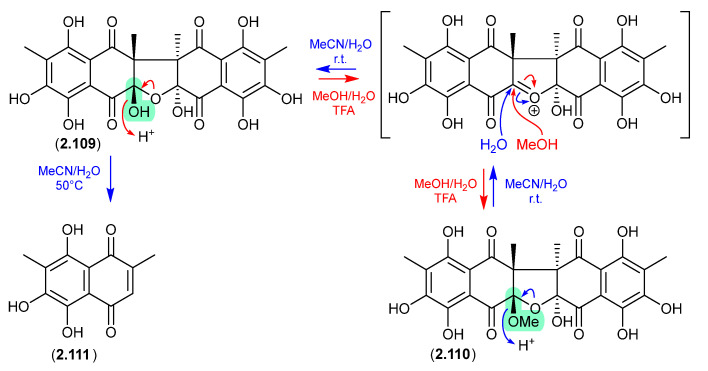
Talcarpones.

### 2.6. Chloroform

#### Greensporones (Figure 30)

A CDCl_3_ solution of the resorcylic acid lactone greensporone D (**2.112**), isolated from the aquatic fungus *Halenospora* sp. G87, underwent conversion via an intramolecular Michael addition to the tetrahydrofuran greensporone F (**2.113**) [52].

**Figure 30:**
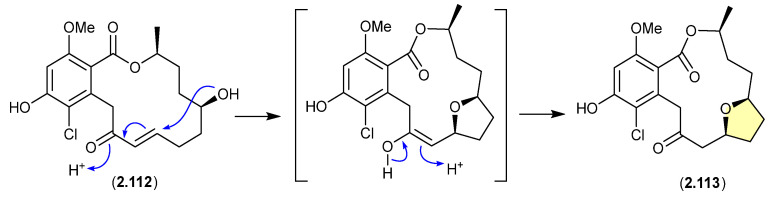
Greensporones.

#### Alkyl Resorcinols (Figure 31)

Alkyl resorcinols isolated from the Chinese soil-derived *Pseudomonas aurantiaca* YM03-Y3, underwent oxidative transformation at r.t. in CDCl_3_ solution, with **2.114** yielding the quinone **2.115**, dimer **2.116**, ring contracted butanolide **2.117** (the latter likely through the oxidative decarboxylation and a range of minor products [53].

**Figure 31:**
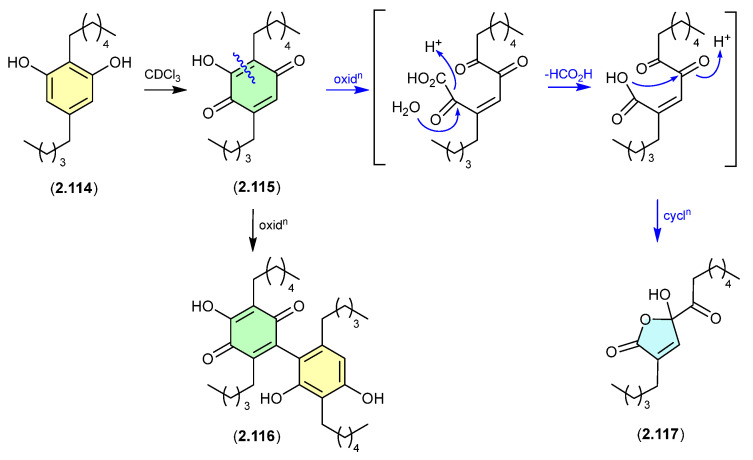
Alkyl Resorcinols.

#### Azodyrecins (Figure 32)

On prolonged storage (30 d) at r.t., a CDCl_3_ solution of the soil-derived *Streptomyces* sp. P8-A2 azoxy compounds, azodyrecins A–C (**2.118**–**2.120**), underwent quantitative double bond isomerization to the *E*-isomers, 1′-*trans*-azodyrecins A–C (**2.121**–**2.123**) [54].

**Figure 32:**
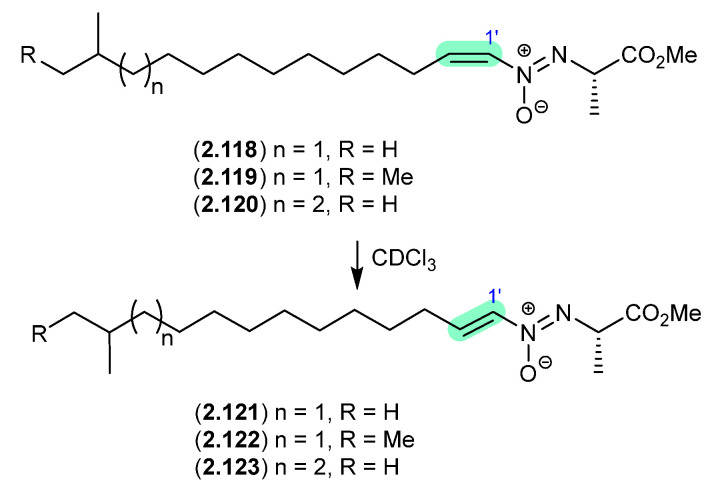
Azodyrecins.

#### Schipenindolenes (Figure 33)

The Chinese fungal endophyte *Penicillium* sp. DG23 yielded indole-diterpenes with HMG-CoA reductase-degrading activity, with schipenindolenes E (**2.124**) and I (**2.125**) undergoing an unexpected equilibration when stored for 2 days at r.t. in CDCl_3_ [55].

**Figure 33:**
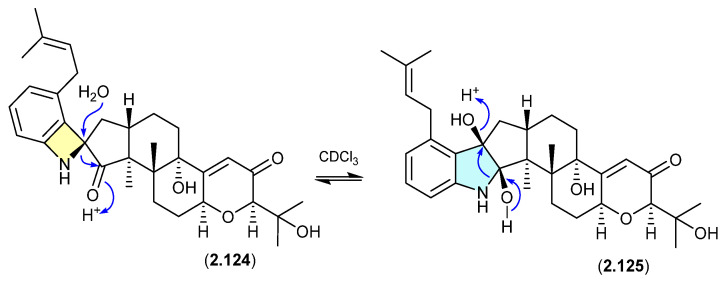
Schipenindolenes.

#### Shearinines (Figure 34 and Figure 35)

A Chinese mangrove-derived *Penicillium* sp. yielded an array of new indolo-terpenes, of which noteworthy examples (relevant to this review) include shearinines A (**2.126**), F (**2.127**), I (**2.128**), H (**2.129**), J (**2.130**) and K (**2.131**) [56]. Significantly, in CDCl_3_ at r.t. shearinine K (**2.131**) partially transformed into shearinine J (**2.130**). As fractionation of the fermentation involved silica gel (CH_2_Cl_2_/MeOH) chromatography, it is likely **2.130** is an artifact of **2.131**. Extrapolating on this, it is also likely that shearinines I (**2.128**) and H (**2.129**) are comparable artifacts of shearinines A (**2.126**) and F (**2.127**), respectively.

The Chinese mangrove rhizosphere soil-derived fungus *Penicillium* sp. N4-3 yielded further examples of the shearinine structure class, including shearinine S (**2.132**), which in CDCl_3_ transformed into the artifact shearinine T (**2.133**) [57]. It has been proposed that this oxidative ring opening is initiated by autoxidation [56,57].

**Figure 34:**
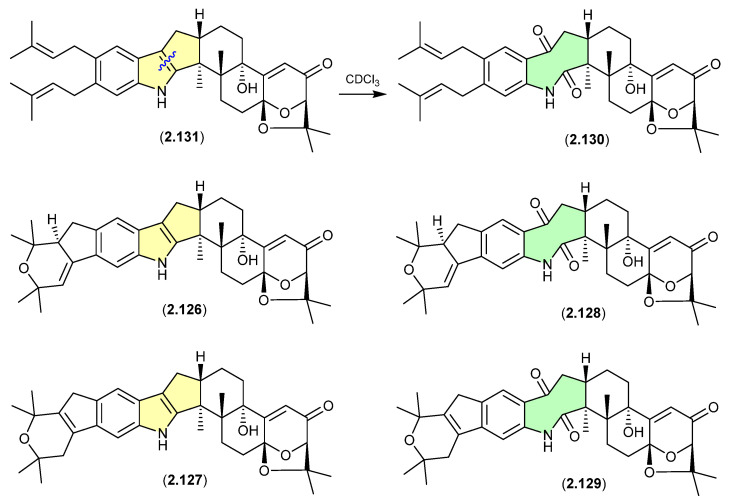
Shearinines.

**Figure 35:**
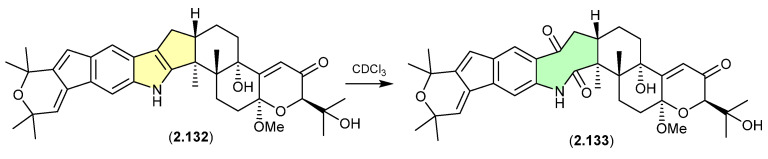
Shearinines.

#### Cavoxin/Cavoxone (Figure 36)

A revision of the structure of cavoxin (**2.134**) (also known as aposphaerin A) produced by the fungus *Phoma cava* reaffirmed that exposure to CDCl_3_ initiates an intramolecular acid-mediated cyclisation to yield the artifact cavoxone (**2.135**) [58].

**Figure 36:**
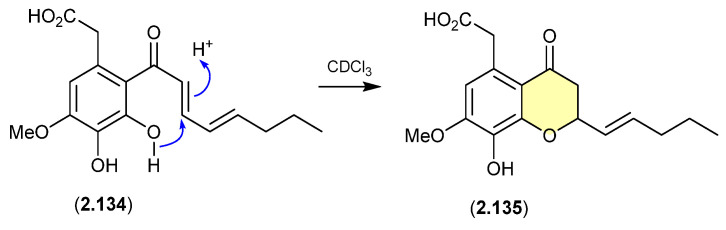
Cavoxin/Cavoxone.

#### Pyrrolizin-3-ones (Figure 37)

The marine-derived *Streptomyces* sp. QD518 yielded the azocin-2-one **2.136**, which during NMR (CDCl_3_) data acquisition underwent complete transformation to **2.137** and **2.138** [59]. A plausible ring rearrangement mechanism is proposed below.

**Figure 37:**
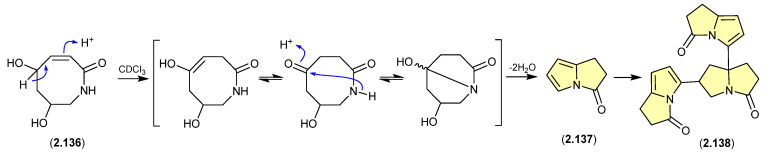
Pyrrolizin-3-ones.

### 2.7. Dichloromethane

#### Bromotyramines (Figure 38)

Bromotryptamines recovered from a tropical southwestern Pacific Ocean collection of the marine sponge *Narrabeena nigra* included the *N*-chloromethyl **2.139**, which would have been formed as an artifact during exposure to CH_2_Cl_2_ [60].

**Figure 38:**
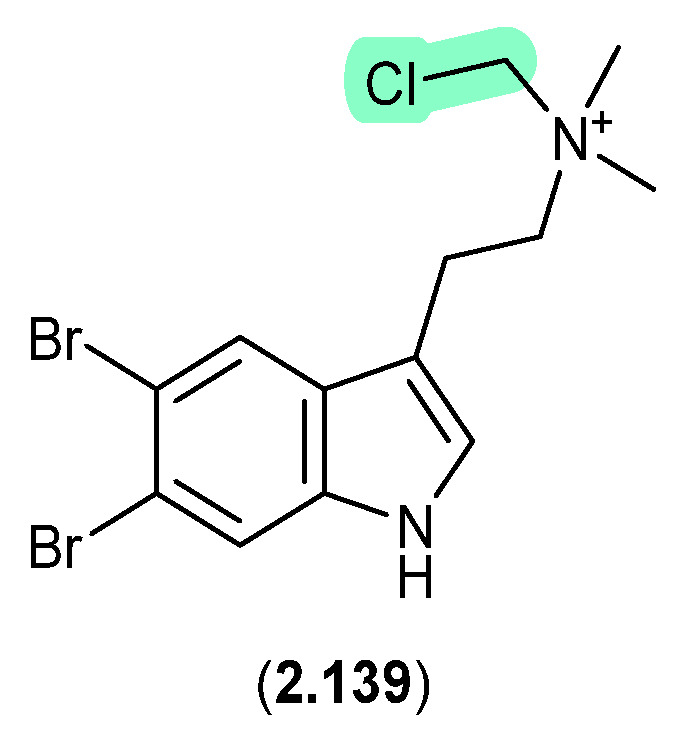
Bromotyramines.

### 2.8. Benzene

#### Theonellastrols (Figure 39)

Oxygenated sterols from a Philippines marine sponge *Theonella swinhoei* included 8α-hydroxytheonellasterol (**2.140**), which in benzene-*d*_6_ at r.t. underwent spontaneous allylic hydroxy migration to the artifact 15α-hydroxytheonellasterol (**2.141**) [61].

**Figure 39:**
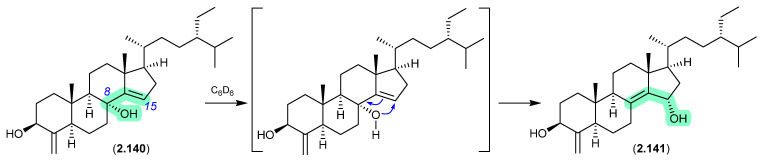
Theonellastrols.

### 2.9. Ethyl Acetate

#### Sorbicillinol/Sorbivetone (Figure 40)

The new sorbicillinoid sorbivinetone (**2.142**) (also known as rezishanone C) isolated from the sponge-derived fungus *Penicillium chrysogenum* DSM 16137 featured a biosynthetically unusual ethoxy moiety [62]. This prompted speculation that **2.142** could be a Diels–Alder cycloaddition artifact of ethyl vinyl ether (potentially an impurity in EtOAc) and sorbicillinol (**2.143**)—the latter a highly reactive natural product and precursor of many sorbicillin-derived fungal metabolites, known to readily undergo Diels–Alder reactions [63]. Supportive of this hypothesis, [^13^C_2_]-acetate incorporation studies revealed that neither the ethoxy moiety nor the two bridging C-atoms were labelled. Furthermore, a dilute aqueous MeCN solution of ethyl vinyl ethyl and the more stable *O*-acetylsorbicillinol (**2.144**) yielded the predicted Diels-Alder adduct, *O*-acetylsorbivinetone (**2.145**).

**Figure 40:**
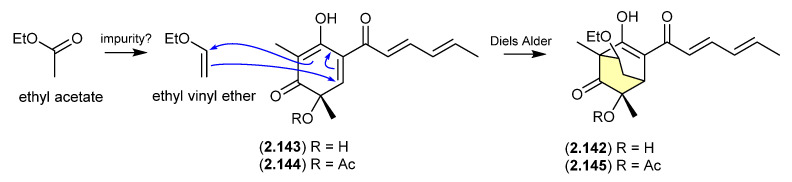
Sorbicillinol/sorbivetone.

### 2.10. Aprotic vs. Protic

While not strictly artifacts, the following examples reveal how natural products can change structures in response to the solvent they are dissolved in—in particular between polar protic versus less polar aprotic solvents. This has particular relevance when exploring SAR and in attempting molecular docking analyses of binding interactions with putative molecular targets.

#### Oxandrastins (Figure 41)

The Australian fungus *Penicillium* sp. CMB-MD14 yielded an array of meroterpenes, exemplified by oxandrastin A (**2.146**), where ring D exists in two solvent-dependent tautomers, as evident in the NMR (CDCl_3_) and NMR (methanol-*d*_4_) spectra [64].

**Figure 41:**
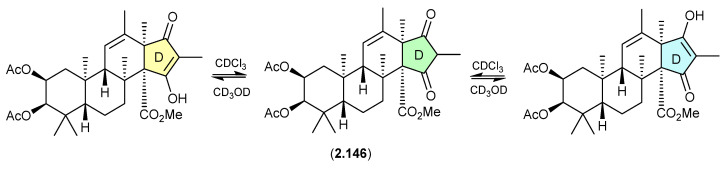
Oxandrastins.

#### Alaeolide (Figure 42)

The Japanese marine sediment-derived *Streptomyces* sp. NPS554 yielded the polycyclic polyketide akaeolide (**2.147**), which exists as two solvent-dependent tautomers—as evident in the NMR (CDCl_3_) and NMR (pyridine-*d*_5_) spectra [65].

**Figure 42:**
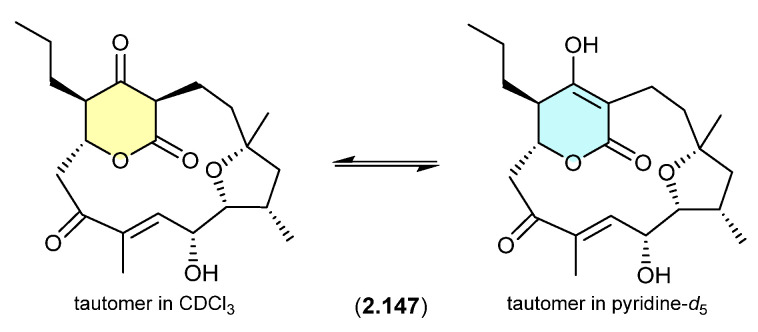
Alaeolide.

#### Pratensilins/Pratenones (Figure 43 and Figure 44)

A 2017 account of the Chinese marine sediment-derived *Streptomyces* sp. KCB-132 reported three spiro indolinone–naphthofuran enantiomeric pairs, (+)-(*S*)-pratensilin A (**2.148**) and (−)-(*R*)-pratensilin A (**2.149**); (+)-(*S*)-pratensilin B (**2.150**) and (−)-(*R*)-pratensilin B (**2.151**); and (+)-(*S*)-pratensilin C (**2.152**) and (−)-(*R*)-pratensilin C (**2.153**) [66]. Following resolution by chiral HPLC, the A pair (**2.148**/**2.149**) equilibrated in both MeOH and CH_2_Cl_2_, whereas the B pair (**2.150**/**2.151**) only equilibrated in MeOH, and the C pair (**2.152**/**2.153**) did not equilibrate in either solvent. While the authors propose two mechanisms, both require the loss of naphthalene aromaticity. An alternate mechanism proceeds via collapse of the embedded aminol to an achiral oxonium intermediate, followed by recyclization with scrambling of chirality. The increased level of substitution on the *spiro* lactam nitrogen could explain the lack of reactivity of **2.152**/**2.153**. In addition to retaining naphthalene aromaticity, an oxonium pathway would also benefit from stabilisation by polar solvents and the adjacent electron-rich naphthalene system. As the isolation of the pratensilins involved MeOH extraction followed by silica gel and HPLC with a MeOH eluant, it is possible one of the enantiomers in each pair is an artifact.

**Figure 43:**
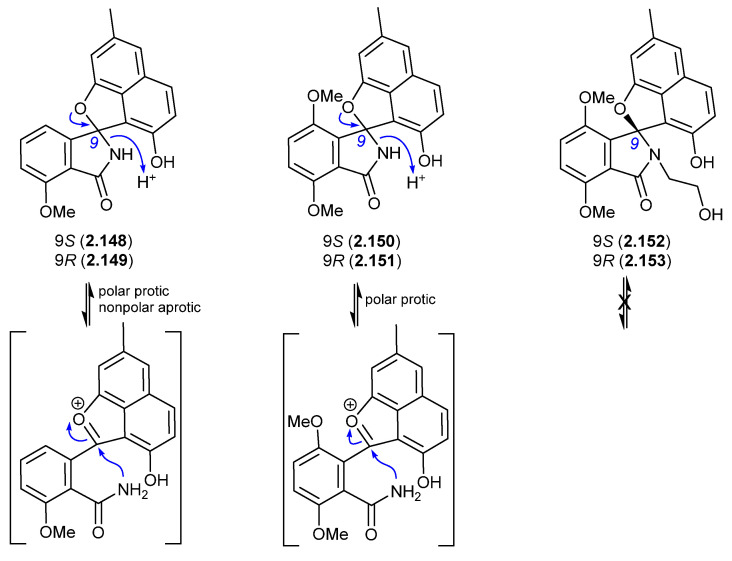
Pratensilins.

In a follow-up 2020 study, *Streptomyces* sp. KCB-132 was also reported to yield another enantiomeric pair, (−)-(*S*)-pratenone (**2.154**) and (+)-(*R*)-pratenone (**2.155**) [67]. Following resolution by chiral HPLC, both enantiomers rapidly racemised (~10 min) in the polar solvents MeOH and THF, but were less prone to racemization in CH_2_Cl_2_ and MeCN. A proposed equilibration mechanism that proceeds via a carbocation (and retains naphthalene aromaticity) would be stabilised by polar over non-polar solvents, and by the electron-rich naphthalene system. As with the pratensilins, the fermentation was initially extracted with MeOH, before being subjected to both silica gel and HPLC with a MeOH eluant, leaving open the possibility that one of the enantiomers is a handling artifact.

**Figure 44:**
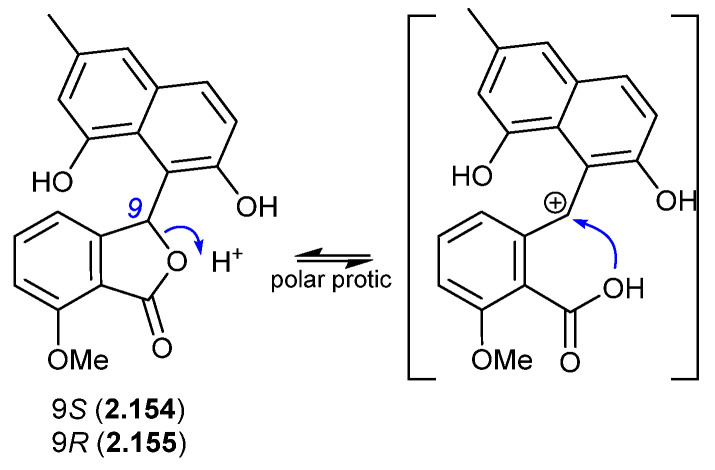
Pratenones.

## 3. Heat

If subjected to elevated temperatures, such as during in vacuo solvent removal from extracts, fractions or pure compounds on a rotary evaporator with a water bath heated to >50 °C, many natural products will decompose (partially or fully), and yet others will transform to artifacts. Consider the following examples.

### 3.1. Psammaplins/Bastadins (Figure 45)

In 1987, Schmitz et al. reported the isomeric oximes, (*E*,*E*)-psammaplin A (**3.1**) and (*E*,*Z*)-psammaplin A (**3.2**) from an unidentified verongiid sponge [68]. This study revealed that on recovery from NMR solvents with mild heating, and subsequent storage over a couple of weeks, **3.2** underwent quantitative transformation to **3.1**—prompting speculation that the true natural product may be the chemically reactive *Z*,*Z* isomer (**3.3**) (see Section 10). The next three decades saw a wealth of reports on structural diverse tyrosine-oxime sponge metabolites—perhaps best exemplified by the cyclic tetrapeptide bastadins common to the genera *Ianthella.* With the literature largely dominated by *E* oxime configurations, in 2010 Crews et al. reported the known (*E*,*E*)-bastadin 19 (**3.4**) along with the new isomer (*E*,*Z*)-bastadin 19 (**3.5**), from a Papua New Guinea collection of *Ianthella* cf. *reticulata* [69]. Of particular note, a 1 h photolysis of (*E*,*E*)-bastadin 19 (**3.4**) in MeCN/MeOH with acetophenone as a sensitiser yielded all possible oxime isomers, namely (*E*,*E*) (**3.4**), (*E*,*Z*) (**3.5**), (*Z*,*E*) (**3.6**) and (*Z*,*Z*) (**3.7**). Likewise, a 30 min sonication (warming) of a DMSO solution of synthetic (*Z*,*Z*) (**3.7**) returned a mixture comprising the full suite of oxime isomers, **3.4**–**3.7**, which on standing in DMSO at r.t. for a further 720 h underwent quantitative conversion to (*E*,*E*) (**3.4**). These observations prompted Crews et al. to speculate that *“…the bastadins and psammaplins initially contain the (Z)-oxime configuration and subsequently isomerize to the more thermodynamically stable E isomer in solution during extraction and/or workup”*, opening up the prospect that a great many sponge oximes reported in the scientific literature were in fact isolation and/or handling artifacts, and that the true (cryptic) natural products have gone unnoticed.

**Figure 45:**
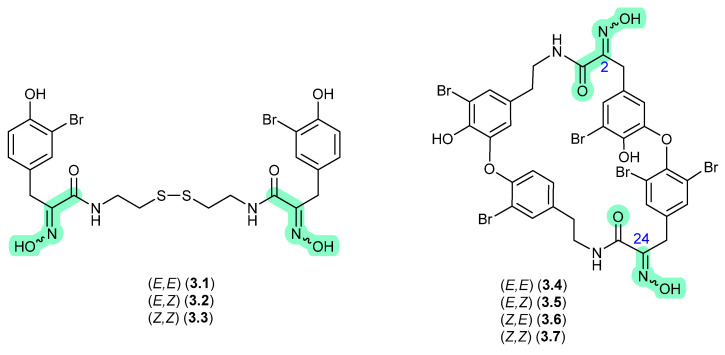
Psammaplins/Bastadins.

### 3.2. Creolophins (Figure 46)

During isolation of the norhirsutane metabolites from culture extracts of the fungus *Creolophus cirrhatus*, creolophin E (**3.8**) was found to be thermally unstable, and on mild heating to 50 °C in vacuo yielded the dimer neocreolophin (**3.9**) through a cascading series of nucleophilic additions and a 1,3-sigmatropic hydride shift [70].

**Figure 46:**
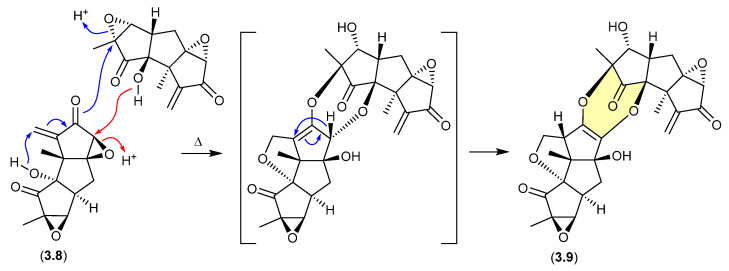
Creolophins.

### 3.3. Neobulgarones (Figure 47)

The Australian fungus *Penicillium* sp. CMB-MD22 yielded a large array of bianthrones as pairs of selectively heat-labile *anti* and *syn* diastereomers, exemplified by (±)-neobulgarone E (**3.10**) and neobulgarone F (**3.11**) [71]. For example, while heating of a MeOH solution of *anti* **3.10** (24 h, 65 °C) led to only 10% conversion to *syn* **3.11**, similar treatment of **3.11** led to complete conversion to **3.10**. Notwithstanding, careful analysis of a fresh fermentation prior to heating or fractionation detected an ~1:1 ratio of all *anti* and *syn* neobulgarones, confirming their status as natural products.

**Figure 47:**
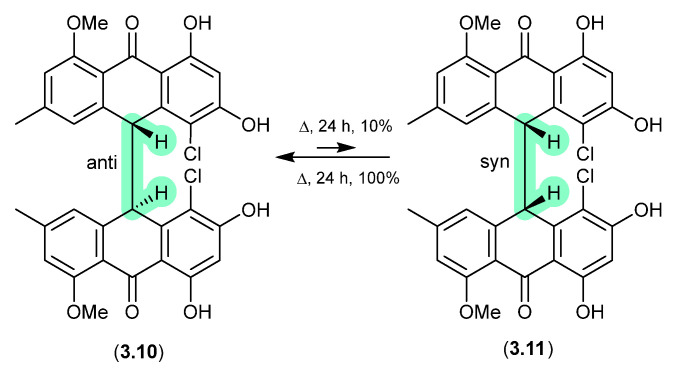
Neobulgarones.

## 4. pH

Many natural products are susceptible to transformation under basic and/or acidic conditions. Variations in pH that occur naturally in fermentation broths, as well as in extracts rich in phenolics or alkaloids, can carry over to organic partitions and be further amplified during in vacuo concentration—particularly as pH-modified aqueous residues are the last to evaporate. Changes in pH can also be introduced either inadvertently or deliberately during fractionation, for example, using acidic media (e.g., silica gel) and/or solvents (e.g., CHCl_3_ or CH_2_Cl_2_), or through the addition of eluant modifiers (e.g., TFA, formic acid, triethylamine (TEA), or various buffers). Variations in pH can also occur during long term storage in solvents that may become acidic over time (e.g., DMSO, CH_2_Cl_2_). Furthermore, as many natural products incorporate acidic or basic functional groups (e.g., phenols, carboxylic acids, amines, guanidines), concentrating/drying-enriched fractions and/or pure samples can facilitate pH-mediated transformations.

### 4.1. Basic

#### Pestalone/Pestalachloride (Figure 48)

The benzophenone pestalone (**4.1**) was first reported in 2001 from the marine brown algae-derived fungus *Pestalotia* sp. CNL-365 (now classified under the genus *Pestalotiopsis*) [72], with a subsequent 2008 report describing the racemic pestalachloride A (**4.2**) from the plant endophytic fungus *Pestalotiopsis adusta* L416 [73]. While racemic natural products are well known, they can nevertheless be indicators of inherent chemical reactivity that enable non-stereo controlled non-enzyme-mediated transformations—much as is the case for artifacts. Interestingly, a 2010 total synthesis revealed that under mild conditions (NH_3_/NH_4_Cl/H_2_O, r.t., 80 min) **4.1** underwent high yield conversion to **4.2** [74]. Of note, the literature inference that **4.1** and **4.2** exist as enantiomeric mixtures of “stable” atropisomers does not seem plausible and lacks experimental validation. It is more probable that **4.1** is achiral, and **4.2** is a racemic mixture of epimers.

**Figure 48:**
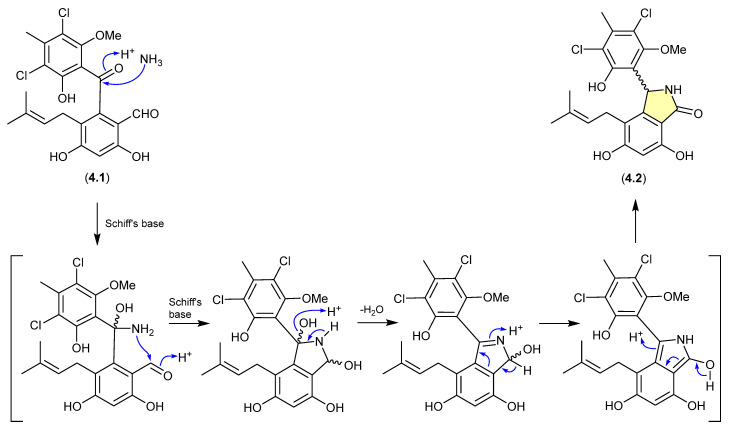
Pestalone/Pestalachloride.

#### Neoenterocins (Figure 49)

Modified M-AM3-D media cultivations of the South China Sea sediment-derived *Streptomyces* sp. SCSIO 11863 yielded the known polyketides 5-deoxyenterocin (**4.3**) and enterocin (**4.4**), and the new neoenterocins A (**4.5**) and B (**4.6**) [75]. Significantly, HPLC analysis of enterocin (**4.4**) in 20 mM PBS buffer (pH 9) yielded **4.6**, and the putative precursor **4.7**, revealing a non-enzymatic pathway from enterocins to neoenterocins.

**Figure 49:**
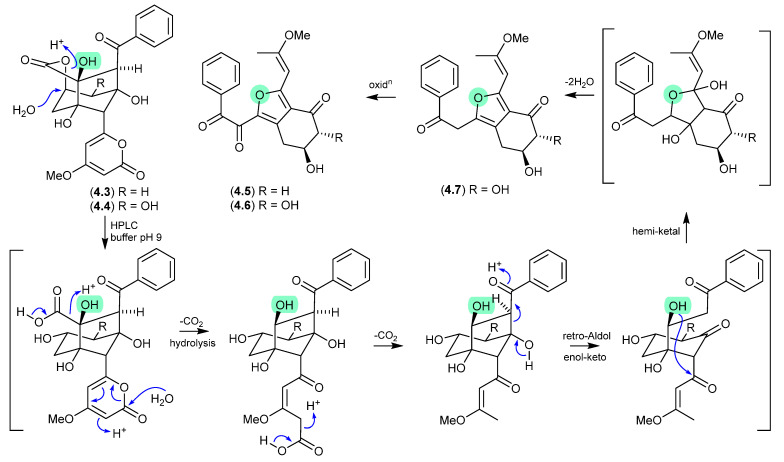
Neoenterocins.

#### Asperazepanones (Figure 50)

The gorgonian coral-derived fungus *Aspergillus candidus* CHNSCLM-0393 yielded the benzoazepine alkaloids, (+)-asperazepanone A (**4.8**), (−)-asperazepanone A (**4.9**) and (+)-asperazepanone B (**4.10**) [76]. While the enantiomers **4.8** and **4.9** could be resolved by chiral HPLC, under mildly basic conditions they rapidly equilibrated to a racemic mixture. The proposed enamine-imino tautomerism mechanism is not available to the *N*-methylated analogue **4.10**, which was isolated as the single (+) enantiomer, suggesting that **4.9** is an artifact.

**Figure 50:**
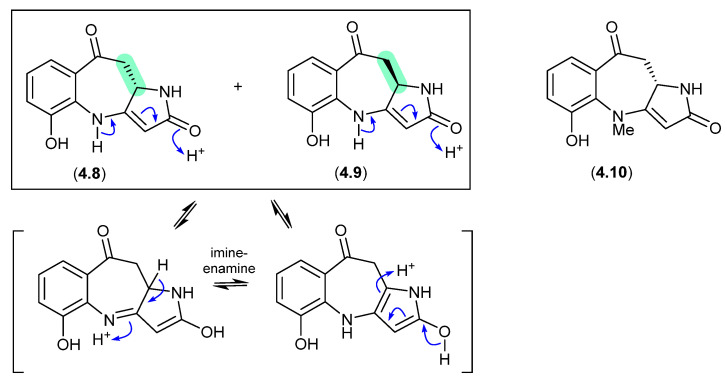
Asperazepanones.

#### Hydroxybrevianamides (Figure 51)

The South China Sea soft coral-derived fungus *Aspergillus* sp. CHNSCLM-0151 yielded two natural products, (+)-17-hydroxybrevianamide N (**4.11**) and (+)-*N*1-methyl-17-hydroxybrevianamide N (**4.12**), each featuring a rare *o*-hydroxyphenylalanine residue [77]. On exposure to mild base, **4.11** and **4.12** formed a racemic mixture with the corresponding epimers **4.13** and **4.14**, respectively.

**Figure 51:**
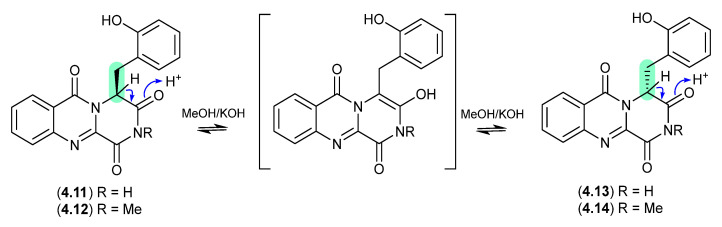
Hydroxybrevianamides.

#### Salinosporamides (Figure 52)

The marine obligate actinomycete *Salinispora tropica* CNB-392 produced the potent proteasome inhibitor salinosporamide A (**4.15**), along with five handling artifacts [78]. These artifacts included the methanolysis products **4.16** and **4.17** (mimicking isolation conditions), and the base-mediated *seco*-decarboxy epimers **4.18**/**4.19**, and its dehydration product **4.20** (mimicking the pH of the fermentation broth).

**Figure 52:**
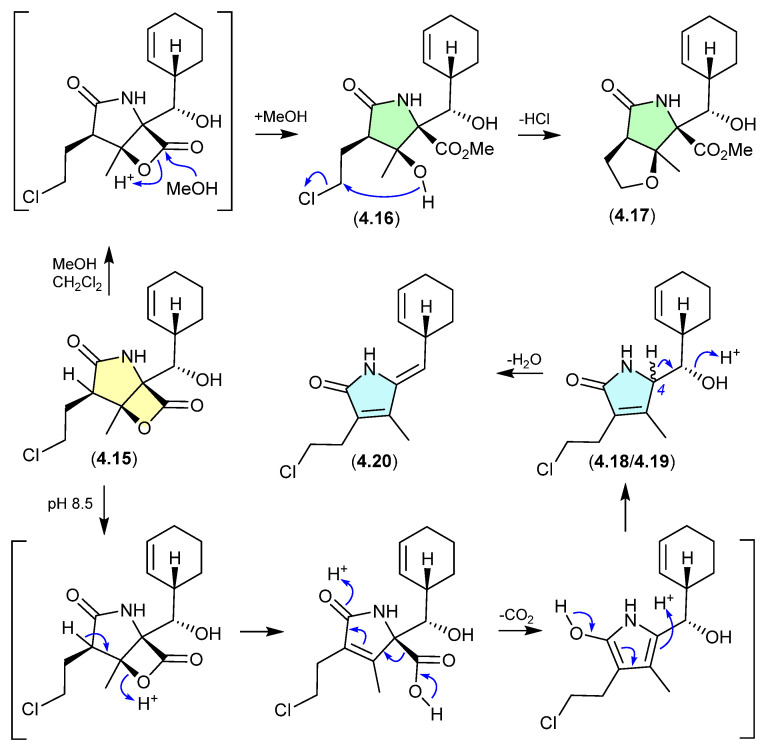
Salinosporamides.

### 4.2. Acidic

#### Enterocins (Figure 53)

The polyketide enterocin (**4.4**) has been reported along with related analogues from a number of sources, including a Western Australian ascidian of the genus *Didemnum* [79], the marine green alga-derived *Streptomyces* sp. OUCMDZ-3434 [80], and the shallow water marine sediment-derived *Streptomyces* sp. BD-26T [81]. A re-isolation of **4.4** from an Australian soil-derived *Streptomyces* sp. CMB-MRB492 prompted the first detailed assessment of its chemical reactivity [82]. When dried under nitrogen at r.t., extracts containing **4.4** proved stable; however, drying with warming to 40 °C resulted in partial conversion of **4.4** to a mixture of enterocins B (**4.21**) and C (**4.22**). Curiously, contrary to the more common role of acid activation of artifact formation, addition of trace amounts of TFA suppressed this thermal transformation. The same could not be said for **4.21** and **4.22**, both of which when stored at r.t. for 24 h in MeCN/H_2_O with 0.01% TFA underwent quantitative conversion to enterocin D (**4.23**). While **4.23** proved stable under long-term storage at low pH, when stored at r.t. for 24 h in MeCN/H_2_O at pH 9 it underwent partial transformation to enterocin E (**4.24**). The pH sensitivity of enterocin (**4.4**) highlights the challenges faced in exploring SAR using cell-based bioassays, where the pH of cultures may vary, even during the course of the bioassay.

**Figure 53:**
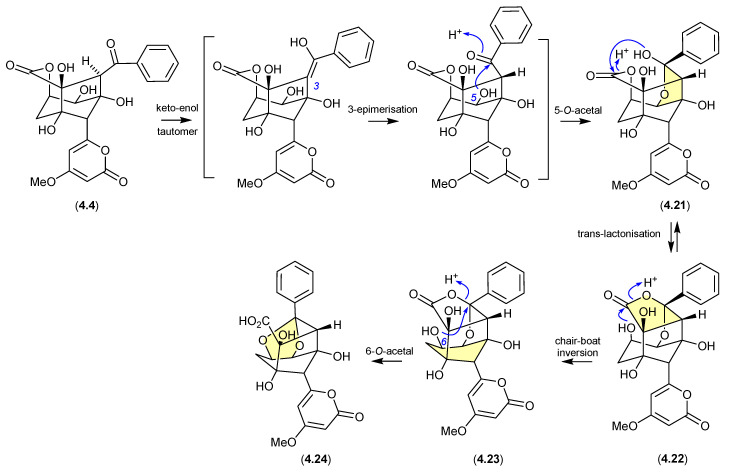
Enterocins.

#### Serratiochelin (Figure 54)

A co-culture of a *Shewanella* sp. and a *Serratia* sp., both sourced as a mixed culture from the intestine/stomach of an Atlantic hagfish (*Myxine glutinosa*) collected by benthic trawl in Hadselfjorden (Norwegian Sea), yielded the known siderophore serratiochelin A (**4.25**) and its formic acid-mediated hydrolysis artifact serratiochelin C (**4.26**) [83]. Interestingly, ring-opened (Thr) compounds such as **4.26** are the biosynthetic precursors of oxazoles such as **4.25**—raising the prospect that the former can potentially be both a natural product and artifact (see Section 12).

**Figure 54:**
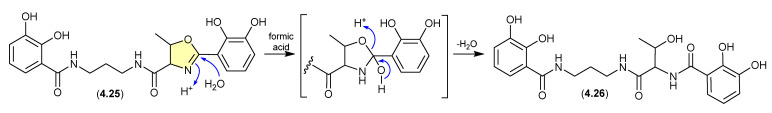
Serratiochelin.

#### Franklinolides (Figure 55)

An Australian marine sponge complex comprising a massive *Geodia* sp. thinly encrusted with a *Halichondria* sp. yielded the exceptionally cytotoxic franklinolide A (**4.27**) [84]. A sample of **4.27** stored at r.t. in methanol-*d*_4_ for several days yielded the deutero-methyl ester **4.28** and the hydrolysed artifact, bitungolide A (**4.29**). The ease of transformation from **4.28** to **4.29** is noteworthy, given that **4.29** was first reported in 2002 from an Indonesian marine sponge, along with an array of geometric isomers, following extensive extraction with MeOH, silica gel chromatography (MeOH/CH_2_Cl_2_) and HPLC (MeOH/H_2_O) [85].

**Figure 55:**
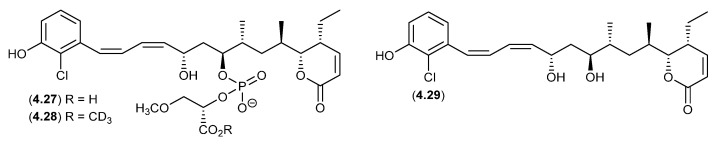
Franklinolides.

#### Oxanthromicins/Eurotones (Figure 56 and Figure 57)

In 2014, the racemic polyketide (±)-*hemi*-oxanthromicin A (**4.30**), isolated from the soil-derived *Streptomyces* sp. MST-134270, was shown to be unstable to acid chromatography (MeOH/H_2_O with 0.1% TFA) [86]. During handling **4.30** transformed into the methanolysis adduct (±)-*hemi*-oxanthromicin A (**4.31**) and the dimeric (±)-*spiro*-oxanthromicin A (**4.32**). Formation of the latter likely proceeds via the co-isolated (±)-*spiro*-oxanthromicins B1/B2 (**4.33**/**4.34**) and C1/C2 (**4.35**/**4.36**), with B1/B2 (**4.33**/**4.34**) transforming during purification (MeOH/H_2_O with 0.1% TFA) to **4.32**.

**Figure 56:**
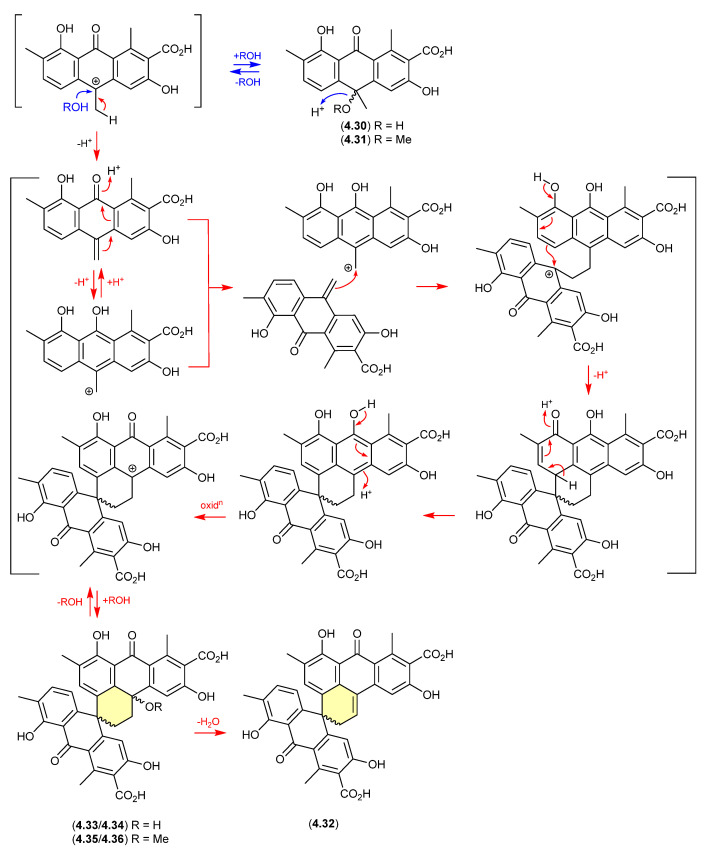
Oxanthromicins.

Curiously, in 2019 the enantiomeric (+)-eurotone A (**4.37**) and (−)-eurotone A (**4.38**) and related monomer, physcion (**4.39**), were reported following silica gel (CHCl_3_/MeOH) fractionation of an extract prepared from the marine-derived fungus *Eurotium* sp. SCSIO F452 [87]—where **4.37**/**4.38** may be indicative of a chemically reactive and cryptic *hemi*-quinone co-metabolite akin to **4.33/4.34**.

**Figure 57:**
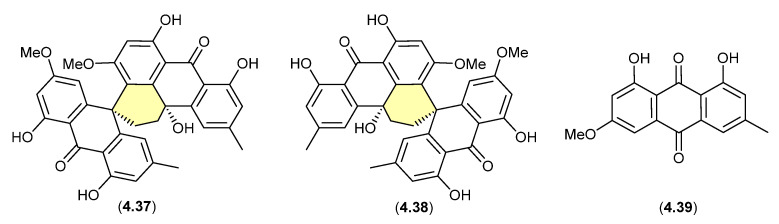
Eurotones.

### 4.3. Silica Gel

#### Sphydrofurans (Figure 58)

On exposure to silica gel chromatography (CHCl_3_/MeOH), the *Streptomyces* metabolite sphydrofuran (**4.40**) transforms into the furan artifact (**4.41**) [88].

**Figure 58:**
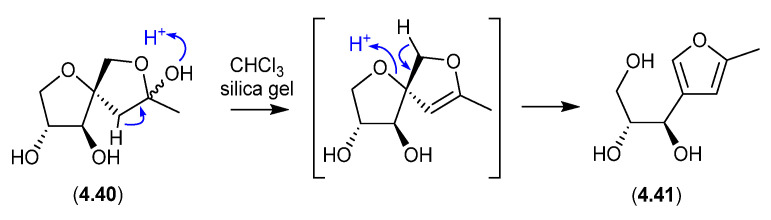
Sphydrofurans.

#### Duclauxin/Bacillisporins/Xenoclauxin/Talaromycesone B (Figure 59 and Figure 60)

Since the polyketide duclauxin (**2.22**) was first reported in 1965 from *Penicillium duclauxii* [89], in excess of 50 analogues have been reported as natural products from both terrestrial and marine fungi of the genera *Penicillium* and *Talaromyces*—many with a wide range of promising biological properties. Over the course of these studies it has been noted/speculated that some duclauxin-*like* natural products (co-metabolites of **2.22**) may be handling artifacts. Recent investigations into the Mexican soil-derived *Talaromyces* sp. IQ-313 [49], examined duclauxin (**2.22**) and the artifact status of the known natural products, talaromycesone B (**4.42**), bacillisporin G (**4.43**), xenoclauxin (**4.44**), and bacillisporins F (**4.45**/**4.46**), J (**4.47**/**4.48**) and I (**4.49**/**4.50**). For example, a solution of **2.22** in DMSO/H_2_O transformed over 24 h to the acetal epimer bacillisporins J (**4.47**/**4.48**), which on dissolution in MeOH underwent spontaneous transformation to bacillisporins F (**4.45**/**4.46**). Similarly, **2.22** adsorbed on silica gel transformed into talaromycesone B (**4.42**); a mixture of **2.22** and **4.47**/**4.48** adsorbed on silica gel transformed into bacillisporin G (**4.43**); a CH_2_Cl_2_ suspension of bacillisporins J (**4.47**/**4.48**) on silica gel generated xenoclauxin (**4.44**); and exposure of **4.47**/**4.48** to mild acid (0.25 N HCl) effected hydrolysis of the acetate moiety to yield bacillisporins I (**4.49**/**4.50**). These studies supported the view that other members of the duclauxin family of natural product are also handling artifacts, including the marine-derived fungal verruculosins (see Section 2.5, duclauxin/verruculosins) [50] and adpressins B–F [90], and terrestrial-derived fungal talaroketals A and B [91], talaroclauxins A and B [92], macrosporusones A–C [93] and bacillisporones A, B, D and E [94].

**Figure 59:**
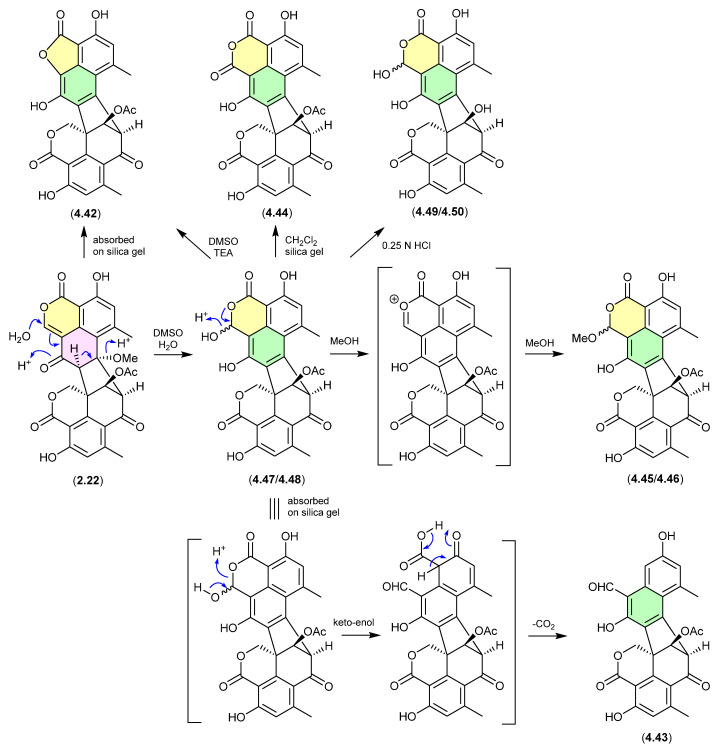
Duclauxin/Bacillisporins/Xenoclauxin/Talaromycesone B.

Duclauxin (**2.22**) has also been shown to react rapidly (in situ during cultivation or extraction—see comment below) with various biogenetically available amines to produce lactams, including with (i) amino acids to yield the talauxins [95], (ii) ethanolamine and γ-aminobutyric acids to yield duclauxamides [96,97] and talaroclauxins [92] and (iii) deoxyaminosugars to yield glyclauxins [18]. While there is no evidence to suggest that these lactams are artifacts, their natural product status does raise an interesting possibility. If the fungus only produces duclauxin (**2.22**), and it is only during solvent extraction that **2.22** is released from the mycelia and comes into contact with biogenic amines present in the culture media—the resulting lactams could be better described as artifacts, as they only come into existence at the point of solvent extraction. This very scenario was encountered in the case of the prolinimines (see Section 10.1).

**Figure 60:**
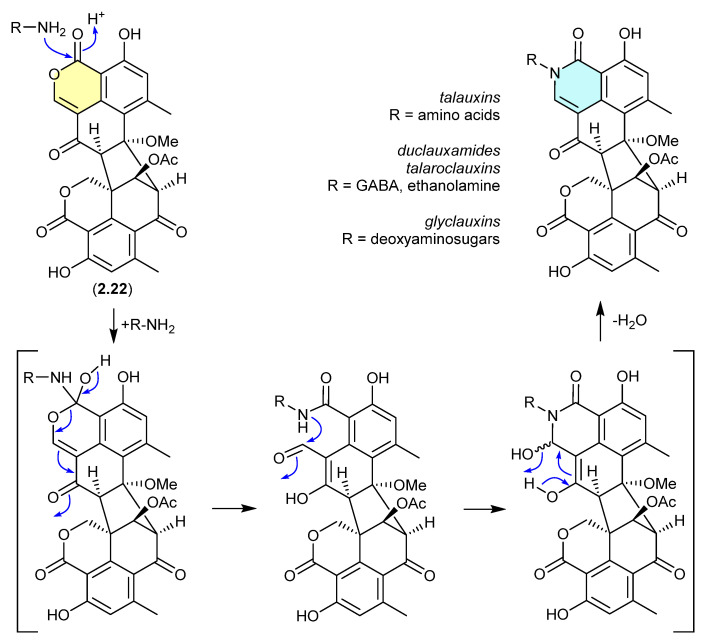
Duclauxin/Talauxins/Duclauxamides/Talaroclauxins/Glyclauxins.

#### Xanthepinone (Figure 61)

The chromone **4.52** recovered from the wood-decay fungus *Rhizina* sp. BCC 12292 was found to be a ring-contracted artifact, induced by silica gel chromatography (MeOH/CH_2_Cl_2_) of the known co-metabolite xanthepinone (**4.51**) [98].

**Figure 61:**
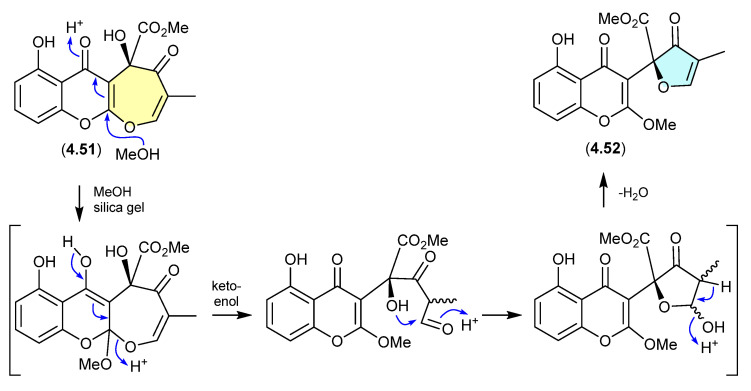
Xanthepinone.

#### Daldinones (Figure 62)

Daldinone H (**4.53**), isolated from the Cameroon mangrove plant-derived fungus *Annulohypoxylon* sp., underwent rapid dehydration on exposure to silica gel chromatography (CH_2_Cl_2_/MeOH) to the artifact daldinone I (**4.54**) [99].

**Figure 62:**
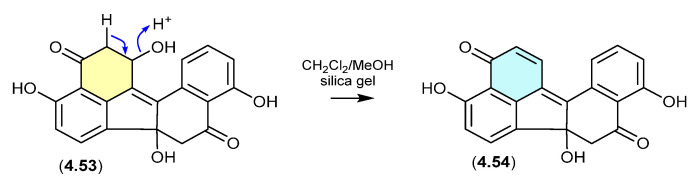
Daldinones.

## 5. Light

Many natural products are sensitive to sunlight, prompting transformations that range from simple changes in configuration through to exquisite rearrangements. It is advisable to minimise exposure to direct sunlight and store them for longer in the dark.

### 5.1. Photoisomerization

#### Pyranpolyenolides (Figure 63)

The South China Sea sediment-derived *Streptomyces* sp. MS110128 yielded two new polyene macrolides that were prone to photoisomerization. For example, after only 5 h exposure to natural light, pyranpolyenolide B (**5.1**) underwent double bond isomerisation to pyranpolyenolides D (**5.2**), E (**5.3**) and F (**5.4**) [100].

**Figure 63:**
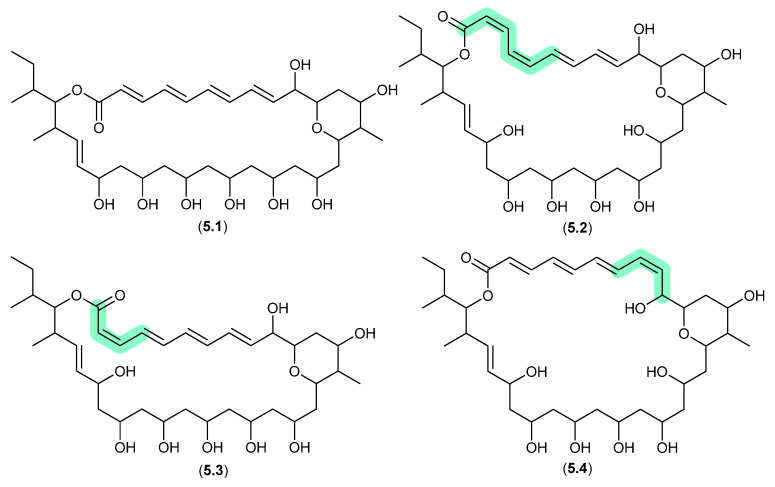
Pyranpolyenolides.

#### Photopiperazines (Figure 64)

A Californian marine sponge-derived actinomycete (strain AJS-327) yielded four isomeric diketopiperazines, photopiperazines A–D (**5.5**–**5.8**), that were prone to photoisomerization [101]. For example, following HPLC purification, and even where care was taken to handle samples under dim light conditions, **5.5** equilibrated to a 1:0.2 ratio with **5.7**. The same was also observed for **5.6** and **5.8**. On exposure to long-wavelength UV (365 nm) for 2 h, this ratio adjusted to 1:1.3; however, when stored under room lighting for 6 h the mixture re-equilibrated to the original ratio. Significantly, photoisomerization was restricted to the double bond associated with the Trp, but not the Leu moiety.

**Figure 64:**
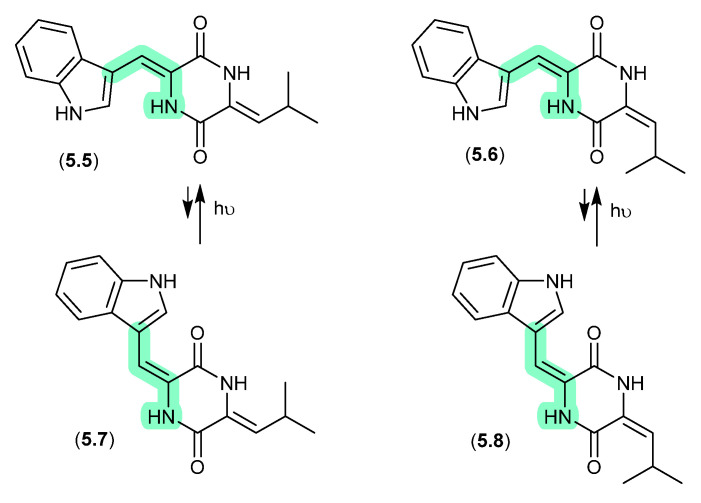
Photopiperazines.

#### Aspochracins/Sclerotiolides (Figure 65)

The halotolerant fungus *Aspergillus sclerotiorum* PT06-1 isolated from salt sediments collected from the Putian Sea Salt Field, Fujian, China, yielded an array of cyclic tripeptides prone to photoisomerism and oxidation [102]. For example, when exposed to daylight and air for 1 d, a MeOH/H_2_O solution of the known cyclic tripeptide aspochracin (**5.9**) underwent isomerisation to sclerotiolide E (**5.10**), and when extended to 10 d, yielded sclerotiolides F–J (**5.11**–**5.16**).

**Figure 65:**
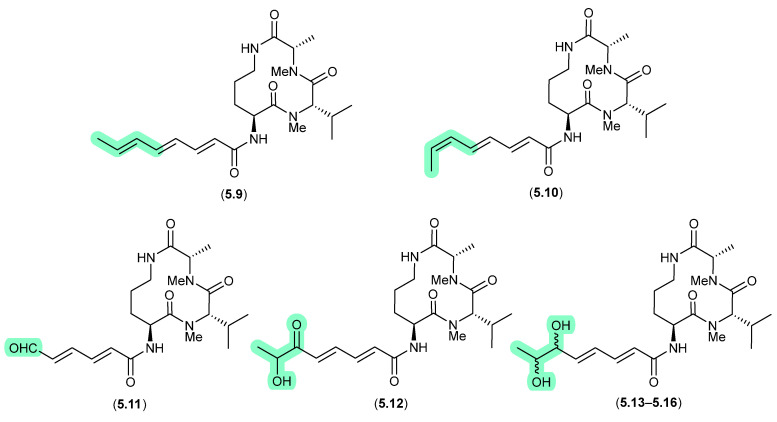
Aspochracins/Sclerotiolides.

#### Clavosines/Calyculins (Figure 66)

A sample of marine sponge *Myriastra clavosa* collected from Chuuk, Federated States of Micronesia, yielded clavosines A–C (**5.17**–**5.19**) as potent cytotoxins and inhibitors of Protein Phosphatase 1 and 2A, of which clavosine C (**5.19**) is a photoisomerization artifact of clavosine B (**5.18**) [103]. Similar issues arose in the case of the structurally related calyculins A–H (**5.20**–**5.27**), where it has been suggested all but calyculins A and C (**5.20** and **5.22**) may be photoisomerization artifacts [104].

**Figure 66:**
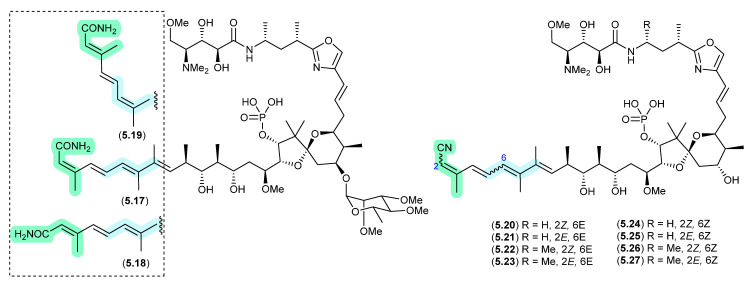
Clavosines/Calyculins.

### 5.2. Photooxidation

#### Cadinanes (Figure 67)

Marine and microbial extracts can be rich in pigments, which could in principle act as photosensitisers, leading to photooxidation artifacts. In an interesting study, Tanaka et al. noted “*We have observed that a neighboring natural products laboratory often isolated molecules with a hydroperoxide moiety, while we have rarely isolated molecules with this functionality. One difference between the two labs is their orientation, with our lab facing north and the neighboring lab facing south. It was therefore suspected that isolation of hydroperoxide molecules could be affected by sunlight coming through windows during laboratory procedures.*” [105] To test this hypothesis, separate acetone, MeCN and MeOH solutions of an isothiocyanate sesquiterpene, cadinane (**5.28**), and the photosensitiser Rose Bengal, were allowed to stand at r.t. for two weeks exposed to sunlight through the laboratory window. The acetone solution yielded **5.29** and **5.30**, while MeCN yielded **5.30**, and MeOH **5.31** and **5.32**. Not only did this study demonstrate the potential for photooxidations, but it also highlighted that photooxidation artifact pathways can be solvent-dependent.

**Figure 67:**
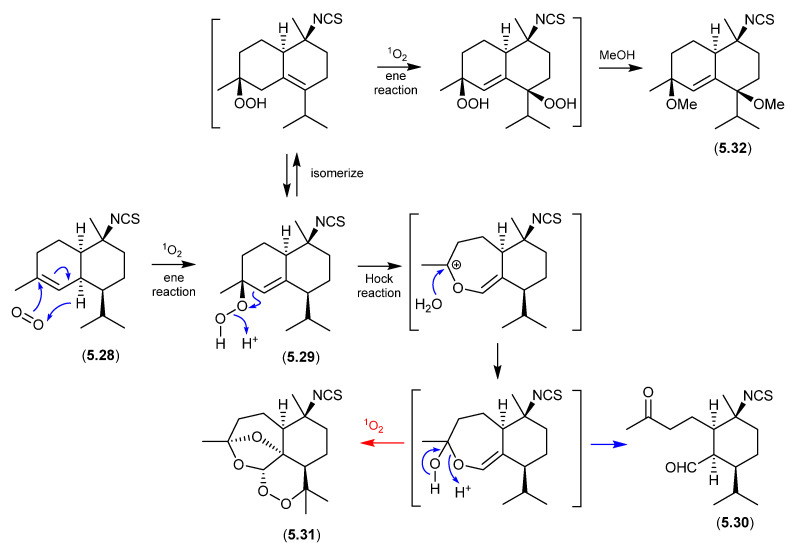
Cadinanes.

### 5.3. Photoreactivity

#### Chetomins (Figure 68)

Chetomin (**5.33**) was first isolated in 1944 by Wakeman et al. from the fungus *Chaetomium cochliodes*, and exhibits strong cytotoxicity via the transcription factor, hypoxia-inducible factor 1 (HIF-1) [106]. Building on this rare structure class, in 2018 Zou et al. reported an investigation of a *C. cochliodes* type strain (CGMCC3.17123), which yielded both **5.33** and the new chetomins A–D (**5.34**–**5.37**) and dethio-tetra (methylthio) chetomin (**5.38**) [37]. As **5.33**–**5.38** proved to be photosensitive, isolation, characterisation and structure elucidation was achieved in darkness. Once isolated, controlled exposure to light indicated a high degree of interconversion between **5.33** and **5.37**, with sulfur heterocycles both enlarging and contracting.

**Figure 68:**
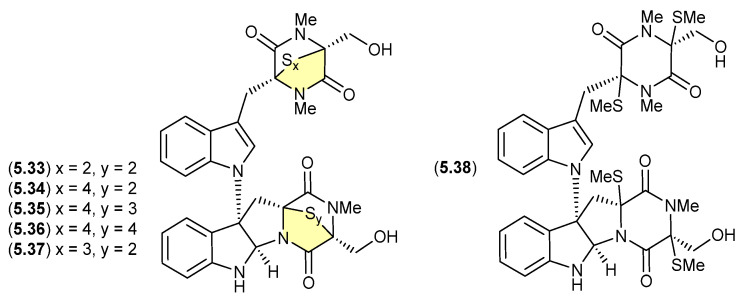
Chetomins.

#### Talaromycins/Purpactins (Figure 69)

The South China Sea gorgonian-derived fungus *Talaromyces* sp. yielded the new diphenyl ether talaromycins A–C (**5.39**–**5.41**), along with several known natural products, including tenellic acid A methyl ester (**5.42**), purpactin C (**5.43**) and C′ (**2.59**) [31]. Interestingly, **5.43** transformed under daylight to **2.59** and **5.39**. Similarly, the extensive use of silica gel (CH_2_Cl_2_/MeOH) chromatography likely facilitated transformation (methanolysis) of the lactone ring in **2.59** to the *seco* methyl ester **5.42**, and the benzaldehydes **5.39** and **5.42**, to the dimethyl acetals **5.40** and **5.41**, respectively (see Section 2.3). Hence, the combination of both sunlight and the use of silica gel suggests that talaromycins A–C (**5.39**–**5.41**), tenellic acid A methyl ester (**5.42**) and purpactin C′ (**2.59**) are likely artifacts.

**Figure 69:**
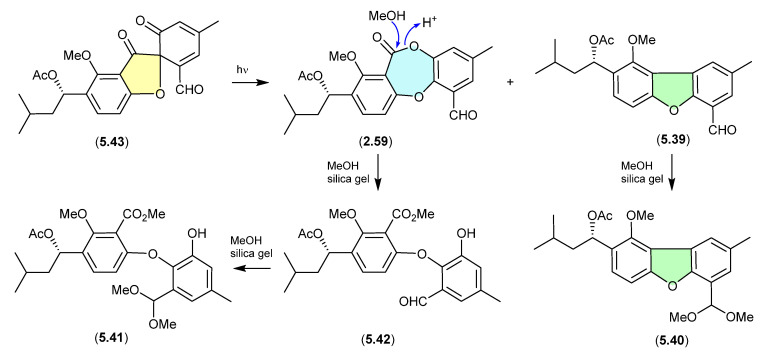
Talaromycins/Purpactins.

## 6. Air Oxidation

Many functional groups are prone to air oxidation, including phenols, hydroquinones, alkenes and alcohols. In many cases, these oxidation artifacts can be readily predicted, and through cautious laboratory handing, be minimised or even avoided. Air oxidation can also initiate transformations that are less intuitive, potentially leading to artifacts that risk going unnoticed and mischaracterised as natural products.

### 6.1. Ketidocillinones (Figure 70)

On exposure to air, the antibacterial polyketide ketidocillinones A (**6.1**) and B (**6.2**), isolated from the Antarctic sponge-derived fungus *Penicillium* sp. HDN151272, slowly oxidised to the corresponding quinones **6.3** and **6.4**, respectively [107]. This is a common transformation among natural products that feature a hydroquinone moiety.

**Figure 70:**
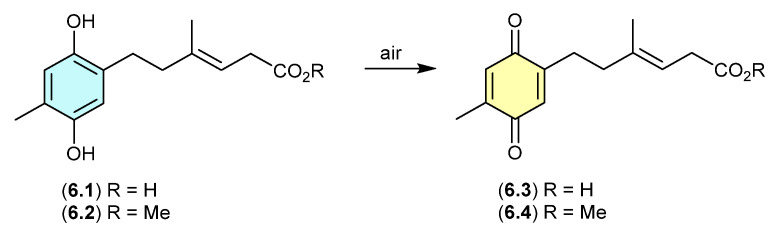
Ketidocillinones.

### 6.2. Pseudopyronines (Figure 71)

The polyketide α-pyrone, pseudopyronine B (**6.5**), isolated from a Fijian marine sponge-derived *Pseudomonas* sp. F92S91, undergoes oxidative transformation during handling to the furanone **6.6** [108]. A key characteristic of this transformation is the racemic nature of the acetal–furanone moiety, and a plausible mechanism proceeds via autoxidation, hydrolysis, decarboxylation/dehydration and lactonisation (as indicated).

**Figure 71:**
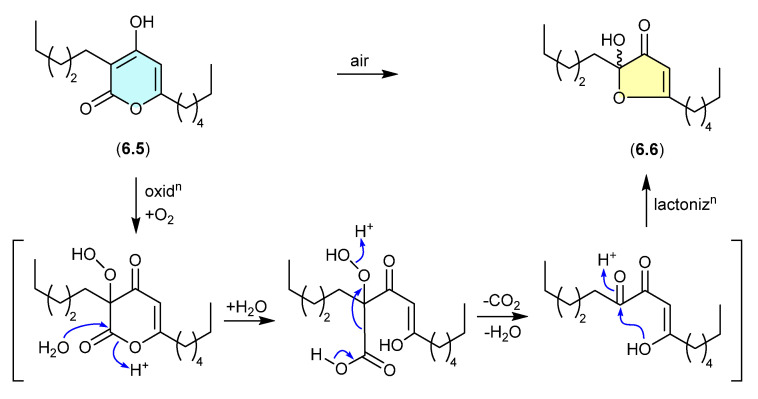
Pseudopyronines.

### 6.3. Norpectinatone (Figure 72)

Natural products featuring the acetal–furanone moiety, as in **6.6**, are relatively rare in the scientific literature, and typically co-occur with the corresponding α-pyrones (e.g., **6.5**), which is suggestive of an artifact relationship. A clear example is seen with the polyketide α-pyrone norpectinatone (**6.7**) and ketal–furanone **6.8** recovered from a Chilean collection of the pulmonated mollusc *Siphonaria lessonii* [109]—the latter potentially an artifact of the former.

**Figure 72:**
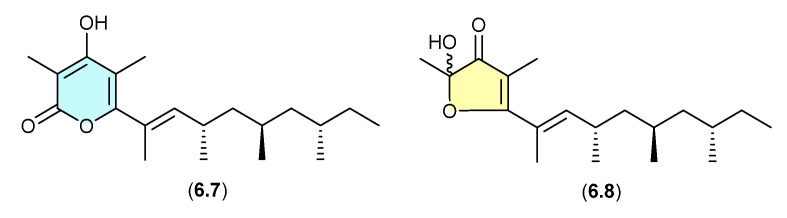
Norpectinatone.

### 6.4. Linfuranones/Hyafurones/Aurafurones (Figure 73)

On other occasions, aggressive cultivation and/or isolation conditions may induce the oxidative biotransformation of prospective (un-isolated) precursor α-pyrones such that only the acetal–furanones are isolated. For example, silica gel fractionation (see Section 4) of extracts prepared from shaken broth (aerated!) cultivations of the actinomycete *Sphaermonospora mesophila* GMKU 363 yielded linfuranones [e.g., linfuranone A (**6.9**)], [110,111] while the myxobacterium *Hyalangium minutum* yielded hyafurones [e.g., hyafurone A_1_ (**6.10**)] [112]. Biosynthetic studies into the closely related myxobacteria aurafurones [e.g., aurafuron A (**6.11**)] provided valuable insights, but did not exclude the possibility of post-translational artifact formation as noted above [113].

**Figure 73:**
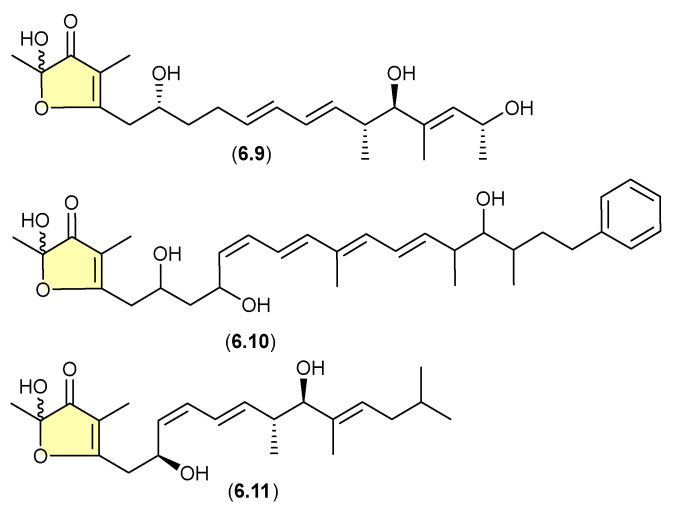
Linfuranones/Hyafurones/Aurafurones.

### 6.5. Avermectins (Figure 74)

Prolonged (multi-year) storage of solid samples of technical abamectin (typically a mixture of avermectin B1a (**6.12**) and minor avermectins), even under refrigeration and without exposure to light, resulted in oxidative transformation (up to 34%) to the remarkably stable and chiral peroxide **6.13** [114].

**Figure 74:**
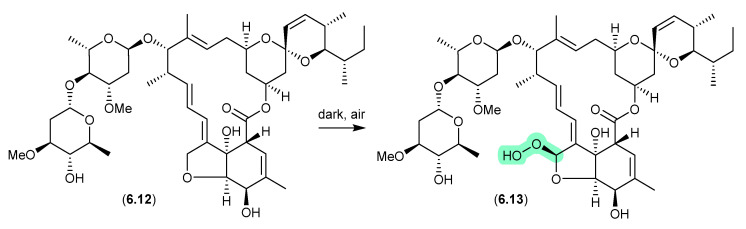
Avermectins.

### 6.6. Penilumamides (Figure 75)

A lumazine-type peptide featuring an l-methionine sulfoxide, penilumamide (**6.14**), was first reported in 2010 from the marine-derived fungus *Penicillium* sp. CNL-338 [115], It was later isolated from the mangrove-derived fungus *Aspergillus* sp. 33,241 in 2015 [116], and from the gorgonian-derived fungus *Aspergillus* sp. XS-20090B15 in 2014 [117]. As the latter study also reported the l-methionine sulfone penilumamide C (**6.15**) as a minor co-metabolite, this prompted speculation that the true natural product may be an as yet undetected cryptic l-methionine analogue **6.16** (see Section 10). This hypothesis was validated when careful examination of an extract of XS-20090B15, avoiding air oxidation, yielded penilumamide B (**6.16**). As predicted, exposure of **6.16** to air at r.t. resulting in its initial transformation to the sulfoxide **6.14**, and later to the sulfone **6.15**.

**Figure 75:**
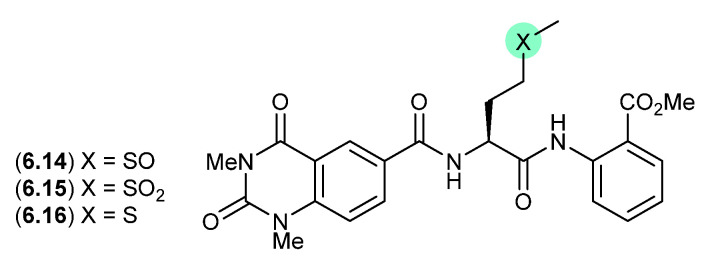
Penilumamides.

## 7. Acetal/Ketal Equilibration

Not strictly a matter of artifacts, acetal/ketal equilibria—much like equilibration in protic versus non-protic solvents (see Section 2.10)—reveals chemical reactivity that can impact on structure elucidation, as well as consideration of biosynthetic origins, SAR, and biological mechanisms of action. For example, failure to understand acetal/ketal equilibria can potentially invalidate in silico docking studies. Likewise, as acetal/ketal equilibria ratios can be influenced by the environment, this complicates quantitative comparison of bioassay data acquired in different systems (i.e., cell-free vs. cell-based assay, in buffers at different pH, and/or in the presence/absence of variables such as metal ions). Many examples of acetal and ketal equilibration are relatively trivial (e.g., carbohydrate anomers), but others are less so. To illustrate this, two case studies are outlined below.

### 7.1. Okichromanone (Figure 76)

Reported in 2024 from the sponge-derived actinomycete *Microbispora* sp., okichromanone (**7.1**) exists as an equilibrating mixture of two racemic ketal regioisomers [118].

**Figure 76:**
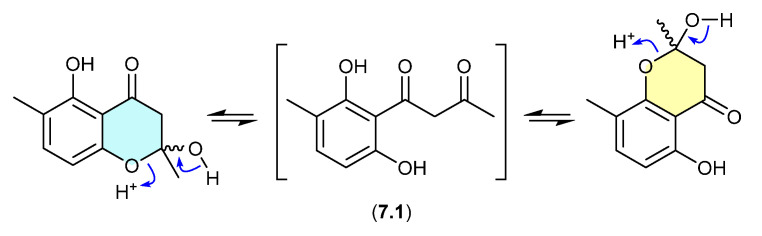
Okichromanone.

### 7.2. Sphydrofuran (Figure 77)

The *Streptomyces* metabolite sphydrofuran (**4.40**) exists as an equilibrating mixture of ketal epimers and the ring-opened ketone (**7.2**), which readily convert in MeOH to a corresponding epimeric mixture of methyl ketals (**4.40**) [88].

**Figure 77:**
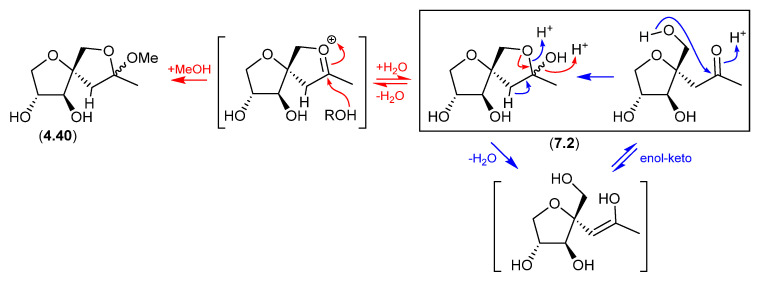
Sphydrofuran.

## 8. *trans*-Esterification

During extraction, fractionation, handling and/or storage, some natural product esters undergo *trans*-esterification. While *trans*-esterifications are not especially common, it is nevertheless a potential pathway to artifact formation.

### 8.1. Kipukasins (Figure 78)

The esterified nucleosides kipukasins M (**8.1**) and N (**8.2**), from the marine-derived fungus *Aspergillus versicolor*, exhibited a facile *trans*-esterification to an equilibrating mixture of regioisomers when stored for 4 h in HPLC solvents [119].

**Figure 78:**
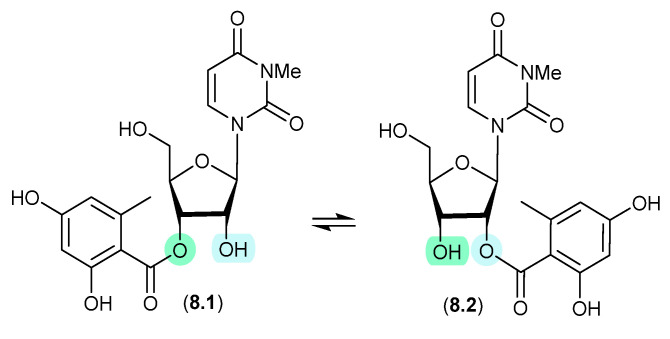
Kipukasins.

### 8.2. Glenthmycins (Figure 79)

On exposure to mild base (K_2_CO_3_ in MeOH), a *trans*-esterification was observed between *Streptomyces* sp. CMB-PB041-derived glenthmycins C (**8.3**), F (**8.4**) and H (**8.5**), and between glenthmycins E (**8.6**), N (**8.7**) and M (**8.8**) [120].

**Figure 79:**
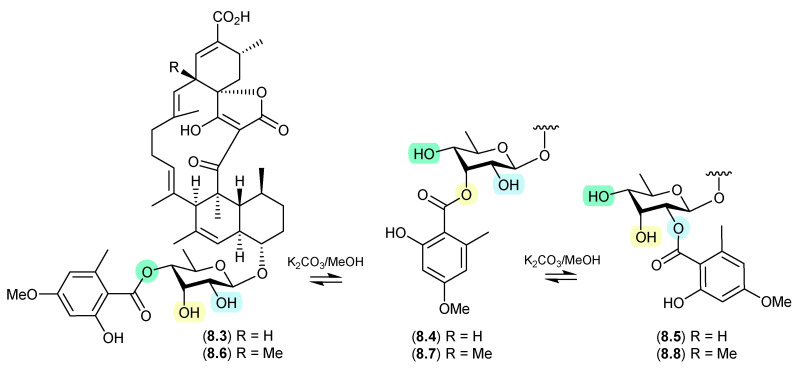
Glenthmycins.

### 8.3. Amaurones (Figure 80 and Figure 81)

The Australian mullet fish gastrointestinal tract-derived fungus *Amauroascus* sp. CMB-F713 yielded an array of polyketide pyrones with unprecedented carbon skeletons, including amaurones A–C (**8.9**–**8.11**), E (**8.12**) and J (**8.13**) [121]. Drying a solution of the orthoacetate **8.12** under nitrogen (at 40 °C overnight) resulted in hydrolysis to a 3:2:1 mixture with the monoacetates **8.10** and **8.11**, which on further heating (4 d) returned a 1:1 mixture of **8.12** and **8.10**, favouring the regioisomer with an equatorially (rather than axially) disposed acetate moiety. A comparable transformation was observed between **8.9** and **8.13**, necessitating chair–chair ring inversion to bring the hydroxy and acetate moieties into a suitable 1,3-diaxial arrangement, where on this occasion both regioisomers featured equatorially disposed acetate groups.

**Figure 80:**
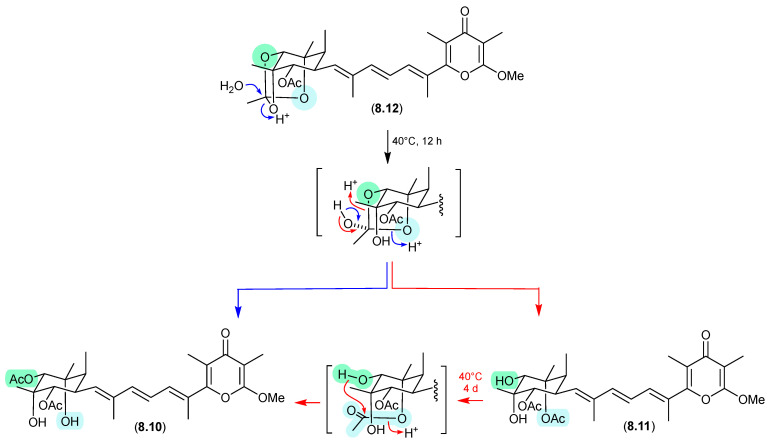
Amaurones.

**Figure 81:**
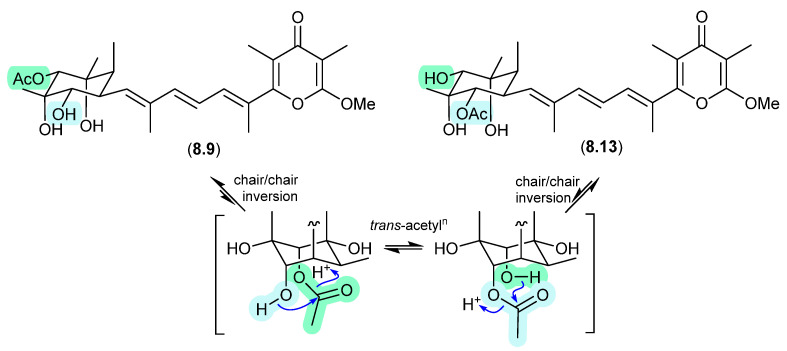
Amaurones.

## 9. Epimerization

In selected natural products, non-acetal chiral centres can undergo epimerisation, providing insights into the chemical reactivity of unusual functionality and/or scaffolds.

### 9.1. Aspergillazines (Figure 82)

Indicative of how even slight changes in functionalisation can have a dramatic effect on chemical reactivity, the epimeric and highly modified *Aspergillus* thiophane dipeptides, aspergillazines B (**9.1**) and C (**9.2**), did not undergo equilibration/epimerization on handling, whereas the corresponding tetrahydrofuran analogues, aspergillazines D (**9.3**) and E (**9.4**), underwent rapid equilibration/epimerization to a 1:0.85 mixture (most likely via a ring-opened imine intermediate) [122].

**Figure 82:**
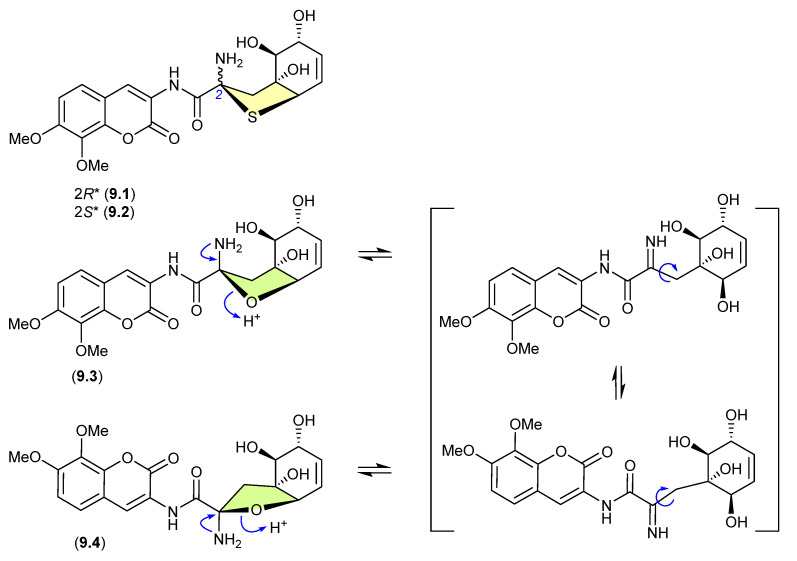
Aspergillazines.

### 9.2. Quinolactacins (Figure 83 and Figure 84)

Planar structures for the tumor necrosis factor (TNF)-inhibitory quinolactacins A–C were first reported in 2000 from an undescribed *Penicillium* sp. [123]. A subsequent 2001 account revealed that quinolactacin A from *Penicillium citrinum* 90684 was a mixture of the epimers A1 (**9.5**) and A2 (**9.6**), with the latter being 14 times more potent at inhibiting acetylcholinesterase [124]. Significantly, the stereochemical difference between **9.5** and **9.6** was asserted at that time (incorrectly) to be about the sidechain 2°-methyl, suggestive of biosynthetic incorporation of Ile and *allo*-Ile. A 2001 biomimetic synthesis of quinolactacin B failed to disclose that it too existed as a mixture of epimers B1 (**9.7**) and B2 (**9.8**) [125], while a 2003 total synthesis confirmed the structure for quinolactacin B2 (**9.8**) but incorrectly affirmed the earlier incorrect stereochemical assignment for A1 (**9.5**) and A2 (**9.6**) [126].

A 2006 investigation into the Australian isolate *Penicillium citrinum* MST-F10130 revealed the true complexity of quinolactacin chemistry and stereochemistry, reporting the known quinolactacins A2 (**9.6**), B2 (**9.8**), C2 (**9.9**) and A1 (**9.5**), as well as the new quinolactacins B1 (**9.7**), C1 (**9.10**), D2 (**9.11**) and D1 (**9.12**), along with the novel analogues quinolonimide (**9.13**) and quinolonic acid (**9.14**) [127]. Significantly, this study revealed that under mild handling conditions, (i) A2 (**9.6**) epimerises to a racemic mixture with A1 (**9.5**), as does B2 (**9.8**) with (**9.7**); (ii) A2 (**9.6**) undergoes a facile oxidation and non-stereospecific H_2_O addition to yield C2 (**9.9**) and C1 (**9.10**); (iii) undergoes oxidation to yield quinolonimide (**9.13**); and (iv) **9.13** undergoes rapid and regiospecific hydrolysis to yield quinolonic acid (**9.14**). The ease with which these epimerisations occurred suggests one of each epimer (i.e., **9.6**/**9.8** or **9.5**/**9.7**) may be a natural product, and their respective epimer a handling artifact. It also seems likely that D1/D2 (**9.11**/**9.12**) are oxidative hydrolysis products of a hitherto undetected quinolactacin bearing a leucyl sidechain (cryptic—see Section 10).

**Figure 83:**
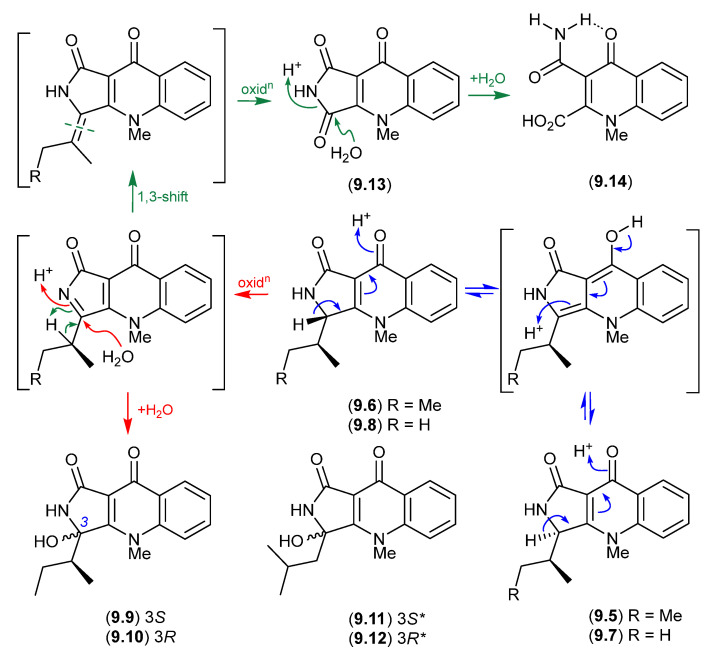
Quinolactacins.

In more recent studies, the known epimeric pairs of quinolactacins A2/A1 (**9.6**/**9.5**), B2/B1 (**9.8**/**9.7**) and C2/C1 (**9.9**/**9.10**), along with the purportedly new epimeric pairs quinolactacins E1/E2 (**9.15**/**9.16**), F1/F2 (**9.17**/**9.18**) and G1/G2 (**9.19**/**9.20**), were reported from a sponge-derived *Penicillium* sp. SCSIO 41303 [128]. Based on the chemical reactivity/artifact studies outlined above, and given the use of silica gel chromatography with a MeOH/CH_2_Cl_2_ eluant, it seems highly probable that (i) E1/E2 (**9.15**/**9.16**) are methanolysis artifacts of the hitherto undetected quinolactacin (cryptic—see Section 10) that is the speculated natural product source of D1/D2 (**9.11**/**9.12**); (ii) F1/F2 (**9.17**/**9.18**) are methanolysis artifacts of the co-metabolites A2/A1 (**9.6**/**9.5**); and (iii) G1/G2 (**9.19**/**9.20**) are hydrolysis artifacts of the co-metabolites B2/B1 (**9.8**/**9.7**). In other words, all the purported new natural products can be rationalised as artifacts.

Similarly, a recent account of an X-ray structure analysis and total synthesis of quinolactacin-H, from a marine-derived *Penicillium* sp. ENP701, revealed it to be a 1:1 magnesium salt of the epimers (*R*)-(+)-quinolactacin-H (**9.21**) and (*S*)-(−)-quinolactacin-H (**9.22**) [129]. As **9.21** and **9.22** are capable of ready equilibration to a mixture of C-3 epimers, it is interesting to speculate whether one, the other or both are natural products. Most recently, in 2023 quinolactacins C1 (**9.10**) and C2 (**9.9**) were reported (incorrectly as new natural products) from the mangrove-derived *Penicillium citrinum* YX-002 [130].

**Figure 84:**
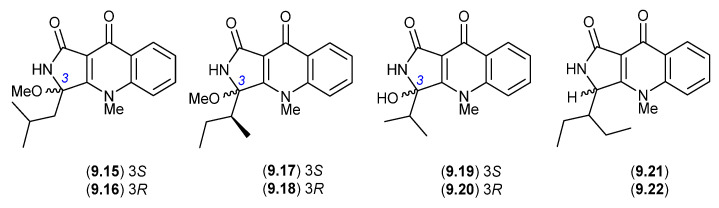
Quinolactacins.

## 10. Cryptic Natural Products

While modern natural products research benefits from highly sensitive technologies that make it possible to analyse chemistry in situ in natural extracts, to fully characterise, understand and explore the potential of natural products requires that they be extracted, purified, characterised, and their chemical and biological properties evaluated experimentally. This can prove very challenging for highly chemically reactive natural products that do not survive extraction and/or handling—a lost opportunity. The following case studies illustrate the value of committing to understanding cryptic natural products.

### 10.1. N-amino-l-proline methyl ester/Prolinimines (Figure 85)

The Australian fish gastrointestinal tract-derived fungus, *Trichoderma* sp. CMB-F563, yielded a series of unprecedented Schiff bases, prolinimines A–D (**10.1**–**10.4**) [131]. Of note, although **10.2**–**10.4** were isolated, characterised and identified from a solvent extract of the fungal culture, **10.1** was not. Nevertheless, it was speculated that **10.1** should be present as a logical biosynthetic precursor of **10.2**–**10.4** and following chemical analysis of fresh extracts it was detected. Moreover, it was demonstrated that during isolation and handling **10.1** underwent rapid and quantitative acid-mediated transformation into **10.3** and **10.4**. The structures for **10.1**–**10.4** were confirmed by detailed spectroscopic analysis and a convergent biomimetic total synthesis starting with *N*-amino-l-proline methyl ester (**10.5**) and 5-hydroxymethylfurfural (**10.6**). These observations revealed **10.1** as a cryptic natural product, with **10.3** and **10.4** designated as artifacts. However, in a surprising twist, a follow-up study aimed at better understanding the biosynthetic origins of the prolinimines revealed that the prolinimines A–D (**10.1**–**10.4)** were artifacts of the “real” cryptic fungal natural product *N*-amino-l-proline methyl ester (**10.5**) [132]. On solvent extraction of the fungal culture, it was shown that **10.5** was released from the fungal mycelia, at which time it came into contact with the media constituent **10.6**—a thermolysis artifact produced during autoclaving of carbohydrate-rich media—and underwent rapid and quantitative transformation into the Schiff bases **10.1** and **10.2**. As noted above, during isolation and handling **10.1** dimerised and trimerised to **10.3** and **10.4**, respectively. The use of culture media depleted in carbohydrates (and hence **10.6**) facilitated the detection and isolation of **10.5**. It is interesting to speculate whether the cryptic small-molecular-weight water-soluble **10.5** would have been discovered without the fortuitous choice of a culture media rich in the thermolysis artifact **10.6**.

**Figure 85:**
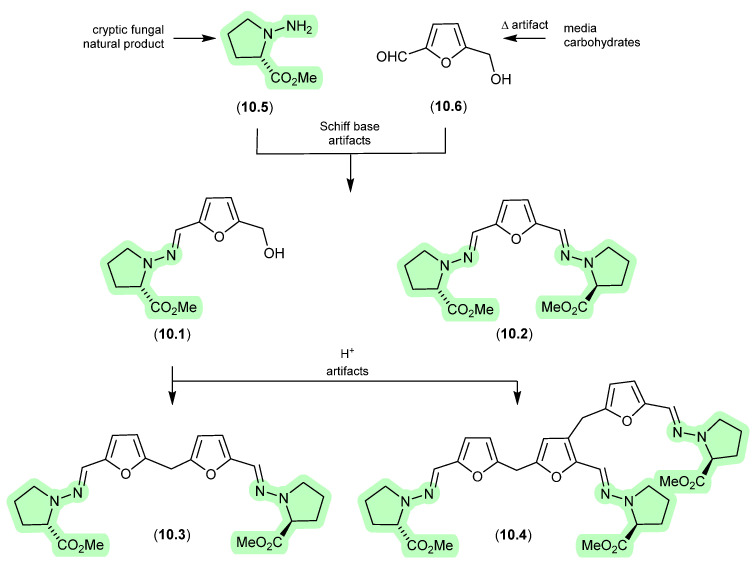
*N*-amino-l-proline methyl ester/Prolinimines.

### 10.2. N-amino-anthranilic acid/Penipacids (Figure 86 and Figure 87)

Re-consideration of the penipacids A–E, first reported as the acyclic amidines **10.7**–**10.11** from the South China deep-sea sediment-derived fungus *Penicillium paneum* SD-44 [133], prompted a total synthesis structure revision as the hydrazones **10.12**–**10.16** [134]. This revision proposed that penipacids A (**10.12**) and B (**10.13**) were Schiff base artifacts of the cryptic (undetected) natural product *N*-amino-anthranilic acid (**10.17**) with diacetone alcohol (**10.18**) and its corresponding methyl ether **10.19**, likely induced by excessive exposure to acetone and methanol under acidic handling conditions. Likewise, penipacids C (**10.14**) and D (**10.15**) were viewed as Schiff base artifacts of **10.17** and the media constituent pyruvic acid (**10.20**) and its methyl ester **10.21**, while penipacid E (**10.16**) was viewed as a Schiff base artifact of **10.17** and the media constituent furfural (**10.22**).

**Figure 86:**
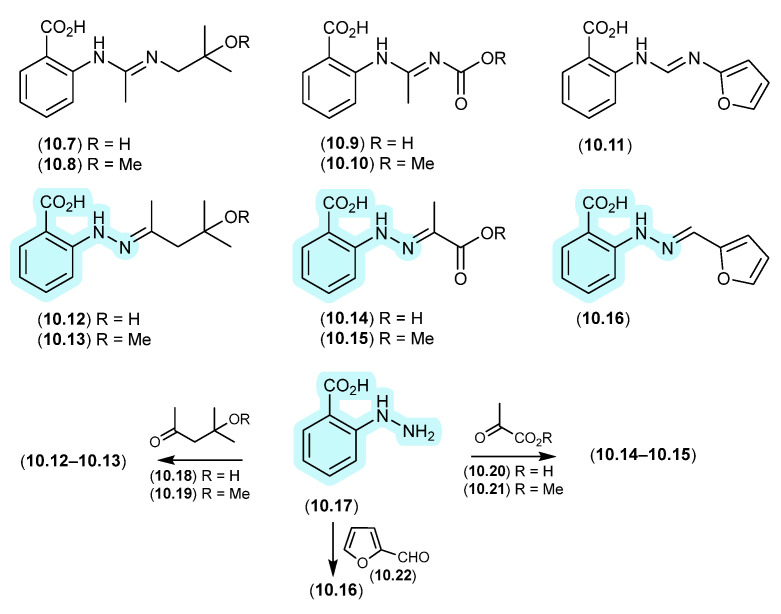
*N*-amino-anthranilic acid/Penipacids.

A review of the natural product literature revealed other instances of potential artifacts based on the purported cryptic natural product **10.17**. These include (i) the γ-glutamylphenylhydrazine anthglutin (**10.23**) as a potential conjugate of **10.17** and L-homoserine from a Japanese fungus *Penicillium oxalicum* SANK 10477 [135]; (ii) the phenylhydrazone farylhydrazones A (**10.24**) and B (**10.25**) from a Tibetan Cordyceps-colonising fungus *Isaria farinosa* as potential Schiff base adducts of **10.17** and pyruvylglycine and pyruvic acid, respectively [136], noting that the revised structure for penipacid C (**10.14**) is now identical to farylhydrazone B (**10.25**); (iii) the 2-azoquinone-phenylhydrazine katorazone (**10.26**) from a Japanese soil-derived *Streptomyces* sp. IFM 11299—a potential Schiff base adduct of **10.17** and the known fungal anthraquinone utahmycin A, which was coincidentally reported to be a co-metabolite with **10.26** [137]; (iv) farylhydrazone C (**10.27**), along with farylhydrazone B (**10.25**), from an Antarctic soil-derived *Penicillium* sp. HDN14-431—as a potential Schiff base adduct of **10.17** with pyruvic acid and dimethylglyoxal, respectively, where the latter is known to be produced during thermal processing of carbohydrate-rich foods, and as such could be a media constituent induced during autoclaving [138]; and (v) the aromatic polyketide murayaquinone C (**10.28**) from the ant gut-derived *Streptomyces* sp. NA4286—a potential Schiff base adduct of **10.17** with a suitably substituted anthraquinone co-metabolite [139].

**Figure 87:**
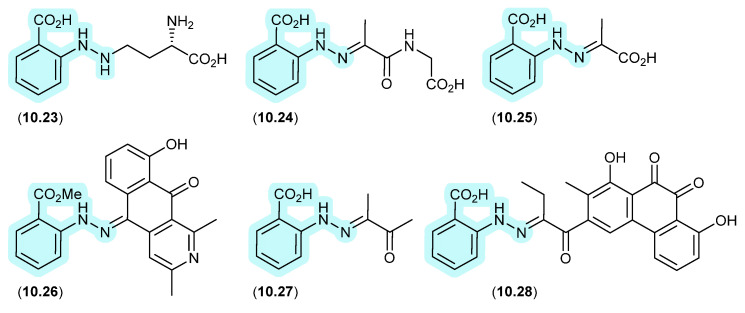
*N*-amino-anthranilic acid adducts.

### 10.3. Elansolids (Figure 88)

In 2011, Müller et al. reported on the atropisomeric elansolids A1 (**10.29**) and A2 (**10.30**), and the *seco* analogues, elansolids B1 (**10.31**) and B2 (**10.32**), as the first polyketide natural products from the non-myxobacterial gliding bacteria *Chitinophaga sancti* [140]. Interestingly, where A2 (**10.30**) exhibited promising antibacterial activity, its atropisomer A1 (**10.29**) was less active. Supportive of structure assignments, on storage in DMSO-*d*_6_ at r.t., A2 (**10.30**) transformed into A1 (**10.29**), while on exposure to a 0.1 M NaOH in MeOH/H_2_O both A2 (**10.30**) and A1 (**10.29**) underwent ring opening to become B2 (**10.32**). This latter observation prompted speculation that B2 (**10.32**) was an artifact of A1 (**10.29**) and A2 (**10.30**) brought about by solvolysis during isolation and handling. In a follow-up study, these authors observed that elansolid production was cultivation condition-dependent, isolated and identified the new seco analogue elansolid D1 (**10.33**), and revealed that B1 (**10.31**) and B2 (**10.32**) were artifacts induced by the addition of H_2_O and MeOH, respectively, to a common chemically reactive cryptic metabolite [141]. With exquisite attention to careful isolation and handling, the cryptic metabolite was successfully isolated and identified as the quinone methide elansolid A3 (**10.34**). Moreover, when exposed to a range of solvents under neutral or mildly acidic or basic conditions, A3 (**10.34**) could transform into all the other elansolids (A1, A2, B1, B2 and D1). Equally, under mildly basic conditions, A1 (**10.29**) and A2 (**10.30**) could transform into A3 (**10.34**). These observations place the chemically reactive quinone methide elansolid A3 (**10.34**) as the pivotal biosynthetic intermediate, and potentially the sole natural product—with other elansolids being artifacts. In a separate study, Piel et al. reported B1 (**10.31**) and the new elansolid D2 (**10.35**) as the sole natural products from another heterotrophic gliding bacterium, *C. pinensis* DSM 2588 [142]. However, Müller et al. demonstrated that on exposure to MeCN/H_2_O (1% TFA)—the HPLC conditions used for the isolation of B1 (**10.31**) and D2 (**10.35**) from *C. pinensis*—A3 (**10.34**) transformed into both D1 (**10.33**) and D2 (**10.35**).

**Figure 88:**
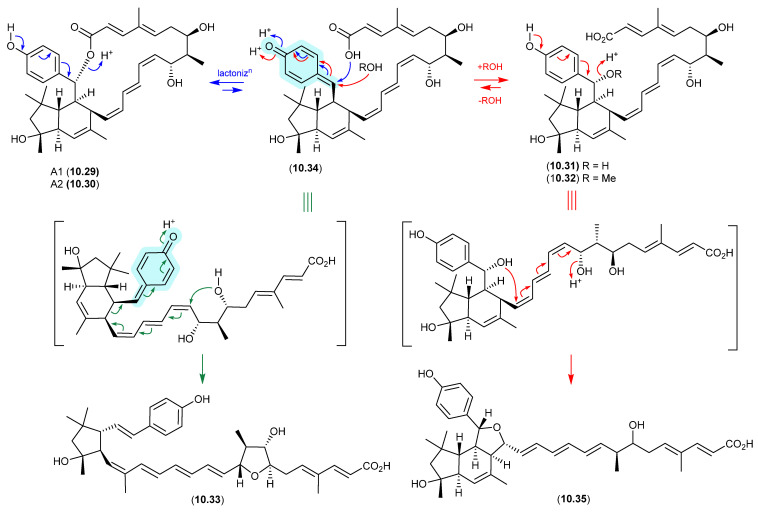
Elansolids.

## 11. Biotransformation

The biological properties of natural products are often determined by applying different concentrations to live cells (e.g., mammalian, bacterial, fungal) and measuring quantifiable changes in cell morphology/viability and/or reporter outputs. Comparison of bioassay data for related natural products reveals SAR, which in turn define pharmacophores and inform our understanding of mechanisms of action. The veracity of any bioassay data relies on the assumption that natural products being tested are stable to assay conditions, and do not convert to other molecules with greater, lesser or even completely different biological properties. While this assumption largely holds true, it is not universally the case. The following case studies illustrate how chemically reactive natural products undergo biotransformation during bioassay.

### 11.1. Abyssomicins (Figure 89)

Abyssomicin J (**11.1**), isolated from the South China Sea deep-sea sediment-derived actinomycete *Verrucosispora* sp. MS100128, was reported to exhibit activity against the bovine tuberculosis bacillus *Mycobacterium bovis* (BCG) [143]. This anti-tubercular activity was unexpected, as earlier SAR studies on the abyssomicin family had pointed to a pharmacophore dependent on an α,β-unsaturated ketone (i.e., a Michael acceptor) [144]. To account for its anti-tubercular properties, it was speculated that **11.1** was a pro-drug, with in situ biotransformation releasing the true anti-tubercular agent. To test this hypothesis, when exposed to a MeCN/H_2_O solution of the oxidising reagent Oxone, **11.1** was reported to undergo rapid oxidation to the sulfoxide **11.2**, which in turn underwent quantitative oxidation to the sulfone **11.3**. Significantly, **11.3** was “primed” for multiple, facile and sequential reverse Michael additions, and on handling yielded the α,β-unsaturated ketone atropisomers, abyssomicin C (**11.4**) and *atrop*-abyssomicin C (**11.5**), with **11.5** being a particularly potent Michael acceptor. Building on these observations, when exposed to BCG cells for only 1 h, **11.1** was reported to undergo rapid (enzymatic) biotransformation to **11.4** and **11.5**. Hence, although not initially apparent, the anti-tubercular activity of **11.1** proved to be dependent on an otherwise cryptic chemical reactivity, and showed propensity for in situ cellular biotransformation.

**Figure 89:**
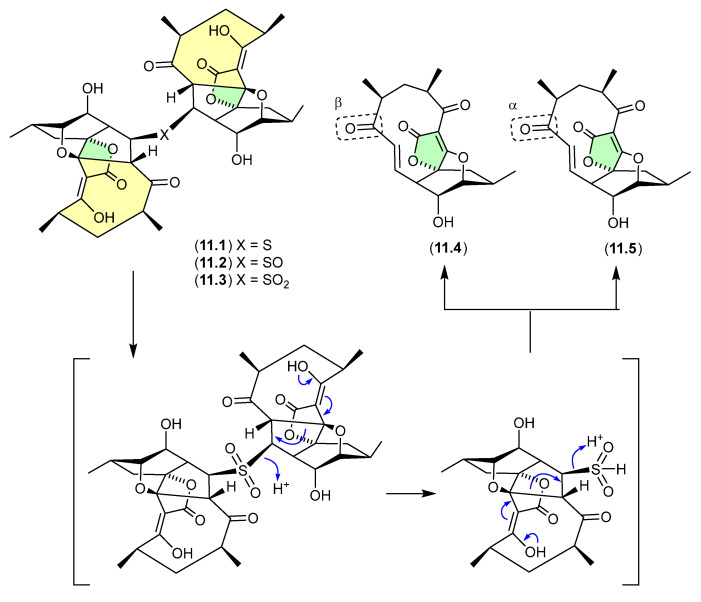
Abyssomicins.

### 11.2. Roseopurpurins (Figure 90)

The Australian estuarine marine mud-derived fungus *Penicillium roseopurpureum* CMB-MF038 yielded an array of polyketides, including the known 15*S*-α,β-dehydrocurvularin (**11.6**) and aculeatusquinone C (**11.7**), and new roseopurpurins H (**11.8**) and I (**11.9**) [145]. Although **11.8** and **11.9** are putative Michael adducts of **11.6** and **11.7**, as they were detected in the crude unfractionated culture extract it was concluded they were natural products—potentially formed in situ by a non-enzymatic Michael addition. More intriguing, while **11.6** exhibited low μM activity (IC_50_) against multiple human cancer cell lines, as would be expected of a Michael acceptor, the same was also true of the supposedly inactivated Michael adducts **11.8** and **11.9**. This prompted speculation that the latter were pro-drugs, prone to reverse Michael addition leading to **11.6**. Indeed, chemical analysis revealed that a 2-6 h exposure to human colon (SW620) and lung (NCI-H460) carcinoma cells was sufficient for quantitative biotransformation of **11.8** and **11.9** to **11.6**. Hence, the cytotoxic activity of **11.8** and **11.9** proved to be dependent on an otherwise cryptic chemical reactivity, and showed propensity for in situ biotransformation.

**Figure 90:**
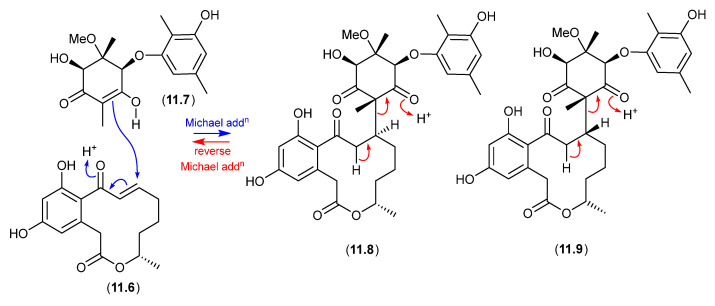
Roseopurpurins.

### 11.3. Kendomycin/Goondomycins (Figure 91)

The Australian pasture soil-derived *Streptomyces* sp. S4S-00052A05 was reported to yield a wide array of polyketides, including the known carbocyclic *ansa*-polyketide kendomycin (**11.10**), and new goondomycins A (**11.11**), B (**11.12**), E (**11.13**) and H (**11.14**)—with all detected in the unfractionated culture extract, confirming their status as natural products [146]. Notwithstanding, overnight exposure of the otherwise chemically stable Michael acceptor **11.10** to *Staphylococcus aureus* cells, resulted in biotransformation to **11.11**–**11.14**. These likely proceed via Michael additions with subsequent oxidations, and further highlight the challenges associated with acquiring meaningful SAR data on chemically reactive natural products.

**Figure 91:**
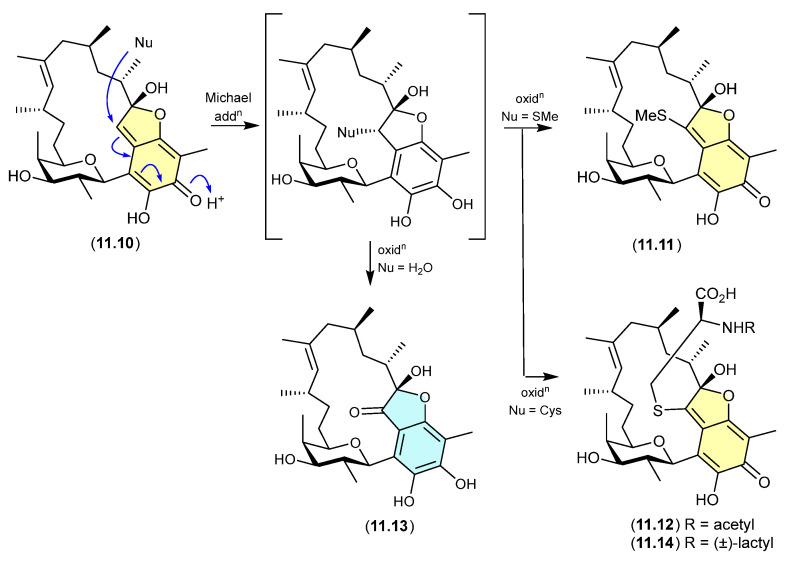
Kendomycin/Goondomycins.

## 12. Conclusions

An awareness of and a willingness to recognise natural product artifacts, as illustrated throughout this review, can be the first step to accessing and understanding the innate chemical reactivity of unique regions of natural product chemical space. We would also encourage some more practical steps that researchers may care to heed.

### 12.1. Practical Workflow to Detect Artifacts

*Be aware*—In the first instance, it is enough to be aware of the various forms of artifacts, and the stimuli that trigger these transformations and the mechanisms behind them. It is also important to be alert to the possibility that any isolated compound may be an artifact. For example, the isolation of acetonides or methyl ethers/esters should raise suspicion if acetone or MeOH was used, and likewise with the presence of an *N*-chloromethyl from a CH_2_Cl_2_ extract, or a dimethyl acetal from a MeOH extract.

*Trial different conditions*—Depending on the nature of the putative artifacts and the extraction and isolation conditions, another strategy for recognising artifacts is to repeat the process using different conditions. For example, if acetone, MeOH or CH_2_Cl_2_ extractions are suspected of generating artifacts, repeat with less reactive solvents (e.g., EtOAc, MeCN).

*Biosynthetic plausibility*—Another useful strategy is to reflect on the likely biosynthetic origin of any isolated compound, as this may reveal structural features that are not obviously natural and may hint at both artifact formation and the likely precursor natural product.

*Monitor fractionation*—At an experimental level, it is also good practice to carefully monitor the fractionation process to detect changes that may inadvertently take place. For example, HPLC analysis of extracts/fractions prior to and after chromatography, especially on silica gel or following the use of acid modified eluants (e.g., TFA), can reveal the appearance of artifacts.

*Establish controls*—Another valuable experimental approach is setting aside a sample of the unfractionated extract as a control. If pure compounds isolated from the fractionated extract are not present in the unfractionated extract, then it is likely they are artifacts.

*Storage artifacts*—Once isolated and identified, samples of natural products (and artifacts) are typically stored either dry or in solution (e.g., DMSO) at low temperature in the dark. These samples can be recovered at a future date and used for further experiments (e.g., [α]_D_, bioassays). While tempting, it is risky to assume that these compounds survive storage intact. It is always prudent to perform a re-analysis (i.e., HPLC-MS) when accessing a stored sample to re-confirm its integrity.

### 12.2. Practical Workflow to Detect Cryptic Natural Products

*Be aware*—Highly chemically reactive cryptic natural products (see Section 10) can be difficult to detect, as they may not survive extraction under normal laboratory conditions. As a result, their transformation artifacts may be isolated, identified and detected in the unfractionated extract, and be miscategorised as natural products.

*Plausible biosynthesis*—One useful strategy is to propose a plausible biosynthesis that accounts for the isolated and identified natural products/artifacts, and see if it predicts the involvement of a potential highly chemically reactive (cryptic) precursor. Forewarning about structure and chemical reactivity can prompt experiments to test for the presence of a putative a cryptic natural product.

*Targeted modification to conditions*—Knowledge of why a natural product is cryptic could prompt removing (or at least minimising) the environmental stimuli. For example, changing pH, solvents, light intensity and/or temperature could enable isolation and structure elucidation.

*Targeted trapping*—Alternatively, understanding why a natural product is cryptic may enable the deliberate addition of “unnatural” precursors or reagents to extracts, to trap the cryptic natural product as a well-defined artifact, thereby unambiguously revealing its presence and structure.

### 12.3. Practical Workflow to Detect Bioassay Biotransformations

*Be aware*—Biotransformation of natural products during bioassays (see Section 11) can generate artifacts in situ which remain undetected and compromise data acquisition and analysis. For example, artifacts may have greater or lesser or even entirely different biological properties, further confounded by the testing of mixtures where the relative ratios may vary over the course of the assay. When carrying out bioassays, always ask yourself—*“Is there any likelihood that this compound may be unstable to the assay conditions?”*

*Chemical analysis of assays*—One option to detect biotransformations during bioassay is to extract the bioassay well at the end of the assay period and perform HPLC-MS or UPLC-MS analysis to identify any potential artifacts. Such analyses should also be accompanied by the extraction and analysis of a control well charged with buffer/media and analyte, but no cells/enzymes. If artifacts are detected in both the assay and control samples, they are likely transformations driven by innate chemical reactivity (i.e., sensitivity to light, temperature, pH, air, etc.). If artifacts are only detected in the assay well, they are probably cell/enzyme-mediated, in which case they are more aptly characterised as biotransformations. The significance of the latter is that the propensity for biotransformation will vary across different cell/enzymes, and this will inevitably complicate quantitative comparisons across different assays. On the other hand, if an analyte is simply chemically labile, it will likely degrade to artifacts in the same way irrespective of the assay (assuming the assay physical conditions are relatively constant), allowing for more confident comparison across assays. This of course does not exclude the possibility that assay outputs are influenced by one or more artifacts, or the possibility that an analyte may undergo both chemical transformations and biotransformations. As a cautionary note, some analytes will form covalent bonds with molecular targets in the assay (i.e., Michael acceptors), so their concentration will diminish with time. As such, the aim of chemical analysis of assays is the detection of potential “confounding” artifacts, not extraction efficiency.

### 12.4. Practical Workflow to Avoid Artifacts

*Solvent extraction*—As noted in Section 2, most extraction solvents bring with them the risk of artifact formation, but some are riskier than others. Particularly problematic solvents include those that are chemically reactive (e.g., acetone, MeOH), and those that can become acidic over time (e.g., CH_2_Cl_2_, CHCl_3_). Two solvents that offer the least risk of artifact formation are EtOAc and MeCN. In addition, care needs to be taken to minimise the presence of solvent impurities, some of which can be chemically reactive (e.g., formaldehyde, acetaldehyde, HCl, peroxides).

*Chromatography*—The classes of chromatography that dominate the modern natural products landscape include silica gel, various forms of reversed-phase MPLC/HPLC (e.g., analytical, semi-preparative and preparative, C8, C18…), and to a lesser degree size exclusion gel (e.g., Sephadex LH-20). That silica gel is inherently acidic is problem enough, but all too often its risk to fragile natural products is compounded by using eluents such as CH_2_Cl_2_, CHCl_3_, MeOH and especially acetone. Silica gel is overwhelmingly the riskiest chromatography media with respect to artifact formation (see Section 4.3). Also of risk is the use of strong acids such as trifluoracetic acid as an eluant modifier in reversed-phase chromatography. While there are occasions where such a modifier is essential for practical resolution, widespread reporting in the natural products, the literature suggests (at least for some laboratories) that its use is the default position, rather than the exception. We would advocate analytical-scale reversed-phase method development with and without a TFA modifier, with follow-up preparative and/or semi-preparative implementation only incorporating TFA when absolutely necessary. Also, where practical, we would advocate MeCN as the organic phase in reversed-phase chromatography over more reactive solvents such as MeOH, CH_2_Cl_2_ and acetone—although clearly the solvent choice will be influenced by solubility issues, and the need for compound resolution.

*pH*—The risk posed by acidic/basic pH can be minimised by careful choice of solvents and chromatography conditions (see above). Notwithstanding, it is important to acknowledge that natural products themselves can feature acidic/basic functionality, and when in concentrated/dry form this functionality can auto-activate pH-mediated artifact formation. For particularly pH-sensitive molecules, auto-activation can be managed by handling/storage in dilute neutral solutions.

*Heat*—It is common to use rotary evaporators equipped with heating baths to remove solvents and concentrate fractions and pure compounds to dryness. Although water baths can operate at high temperatures near 100 °C, and some take advantage of this, we advocate their operation at reduced temperatures (35–40 °C), relying instead on an effective choice of vacuum and condenser chilling to remove solvents. Heat-controlled centrifugal evaporators can also be used for solvent removal.

*Air and light*—While nitrogen purging of flasks/vials and reduced lighting can be a useful precaution when dealing with air/light-sensitive natural products, understandably, unless needs dictate, most researchers carry out operations under normal ambient air and laboratory lighting. That said, as a general rule, it is prudent to avoid exposing any samples to direct sunlight. Likewise, when drying samples under a stream of dry gas, caution dictates the use of nitrogen rather than air.

*NMR data acquisition*—The acquisition of NMR data can take many hours, during which time natural products are exposed to deuterated solvents at r.t. or above. While all the concerns noted above for solvent use in extraction and chromatography apply, it is noteworthy that virtually all NMR solvents have the potential to facilitate artifact formation. Perhaps the only cautionary advice we can offer is to minimise exposure, store samples in the dark at a reduced temperature (~4 °C to avoid freezing and sample precipitation) and avoid exposure to direct sunlight during handling. The same also applies to stored artifacts, as prolonged storage can facilitate further transformations. The authors have encountered cases where the time required to acquire a complete 1D and 2D NMR data set saw a natural product transform—partially or fully—into an artifact. In such cases the early NMR data is acquired on a compound different from the later acquired NMR data.

### 12.5. Why and How to Leverage Knowledge of Artifacts

*Evolved chemical space*—One of the key advantages of studying natural products is that they provide access to unique regions of chemical space that have co-evolved with life on Earth. By mischaracterising artifacts as natural products, we contaminate and dilute our understanding of this chemical space. Similarly, by failing to recognise the artifact status of certain isolated compounds, we risk overlooking valuable chemical knowledge, and in extreme cases (i.e., cryptic natural products) deny ourselves access to some of the most uniquely reactive regions of natural product chemical space.

*Biosynthesis*—Misclassifying artifacts as natural products risks derailing biosynthetic investigations, as ill-informed efforts are made to force a biosynthetic path on compounds that sit outside biosynthetic processes. A similar problem arises when a single natural product is obscured within a sea of artifacts, diluting and misdirecting biosynthetic focus. Conversely, recognising artifacts can yield valuable insights into underlying biosynthetic logic and chemical reactivity.

*Chemical and biological properties*—Knowledge of the relationship between natural products and artifacts can inform the design of highly efficient and convergent biomimetic syntheses, while simultaneously providing insights into chemical stability, which is critical for drug discovery and development. Such knowledge also enables access to greater structure diversity, supporting bioassays and SAR investigations. The development of any natural product as a drug depends upon understanding its chemical properties, which in turn requires an understanding its relationship with artifacts.

## Figures and Tables

**Table 1:** Case studies in marine and microbial natural product artifact formation.

**2. Solvent**	Cavoxin/Cavoxone	Photopiperazines
**2.1. DMSO**	Pyrrolizin-3-ones	Aspochracin/Sclerotiolides
Migrastatins/Dorrigocins	**2.7. Dichloromethane**	Clavosines/Calyculins
Discorhabdins	Bromotyramines	**5.2. Photooxidation**
Dendrillic acids	**2.8. Benzene**	Cadinanes
Methylsulfonated polyketides	Theonellastrols	**5.3. Photoreactive**
Cerulenin	**2.9. Ethyl acetate**	Chetomins
Bisanthraquinones	Sorbicillinol/Sorbivetone	Talaromycins/Purpactins
Glyclauxins	**2.10. Aprotic vs. Protic**	**6. Air oxidation**
Aculeaxanthones/Chrysoxanthones	Oxandrastins	Ketidocillinones
Versixanthones	Alaeolide	Pseudopyronines
Secalonic acid/Parnafungins	Pratensilins/Pratenones	Norpectinatone
**2.2. Pyridine**	**3. Heat**	Linfuranones
Acremoxanthone/Acremonidins	Psammaplins/Bastadins	Hyafurones/Aurafurones
**2.3. Methanol**	Creolophins	Avermectins
Brevianamides	Neobulgarones	Penilumamides
Talaronins	**4. ph**	**7. Acetal/ketal equilibration**
Pyrasplorins	**4.1. Basic**	Okichromanone
Penicipyridones	Pestalone/Pestalachloride	Sphydrofuran
Varacins	Neoenterocins	**8. *trans*-esterification**
Epithiodiketopiperazines	Asperazepanones	Kipukasins
Eleutherobins/Caribaeoranes	Hydroxybrevianamides	Glenthmycins
**2.4. Acetone**	Salinosporamides	Amaurones
Kutzneridines	**4.2. Acidic**	**9. Epimerization**
Enamidonins/K97-0239A and B	Enterocins	Aspergillazines
Autucedines	Serratiochelin	Quinolactacins
Madurastatins	Franklinolides	**10. Cryptic natural products**
Drimanes	Oxanthromicins/Eurotones	*N*-amino-l-proline methyl ester
Duclauxin/Verruculosins	**4.3. Silica Gel**	Prolinimines
**2.5. Acetonitrile**	Sphydrofurans	*N*-amino-anthranilic acid
Talcarpones	Duclauxin/Bacillisporins	Penipacids
**2.6. Chloroform**	Xenoclauxin/Talaromycesone B	Elansolids
Greensporones	Xanthepinone	**11. Bioassay biotransformation**
Alkyl resorcinols	Daldinones	Abyssomicins
Azodyrecins	**5. Light**	Roseopurpurins
Schipenindolenes	**5.1. Photoisomerization**	Kendomycin/Goondomycins
Shearinines	Pyranpolyenolides	

## Data Availability

Not applicable.
